# Endoscopic Study of the Oral and Pharyngeal Cavities in the Common Dolphin, Striped Dolphin, Risso’s Dolphin, Harbour Porpoise and Pilot Whale: Reinforced with Other Diagnostic and Anatomic Techniques

**DOI:** 10.3390/ani11061507

**Published:** 2021-05-22

**Authors:** Álvaro García de los Ríos y Loshuertos, Marta Soler Laguía, Alberto Arencibia Espinosa, Francisco Martínez Gomariz, Cayetano Sánchez Collado, Alfredo López Fernández, Francisco Gil Cano, Juan Seva Alcaraz, Gregorio Ramírez Zarzosa

**Affiliations:** 1 Departamento de Anatomía y Anatomía Patológica Comparadas, Facultad de Veterinaria, Universidad de Murcia, 30100 Murcia, Spain; agrios@ceuta.es (Á.G.d.l.R.y.L.); f.gomariz@colvet.es (F.M.G.); scollado@um.es (C.S.C.); cano@um.es (F.G.C.); jseva@um.es (J.S.A.); 2 Centro de Estudio y Conservación de Animales Marinos (CECAM), 51001 Ceuta, Spain; 3 Departamento de Medicina y Cirugía Animal, Facultad de Veterinaria, Universidad de Murcia, 30100 Murcia, Spain; mtasoler@um.es; 4 Departamento de Morfología, Anatomía y Embriología, Facultad de Veterinaria, Universidad de Las Palmas de Gran Canaria, Trasmontaña, Arucas, 35416 Las Palmas de Gran Canaria, Spain; alberto.arencibia@ulpgc.es; 5 Departamento de Biología—CESAM, Campus Universitario de Santiago, Universidade de Aveiro, 3810-193 Aveiro, Portugal; a.lopez@ua.pt; 6 Coordinadora para el Estudio de los Mamíferos Marinos–CEMMA, Ap. 15, Gondomar, 36380 Pontevedra, Spain

**Keywords:** Striped dolphin (*Stenella coeruleoalba*), Common dolphin (*Delphinus delphis*), Pilot whale (*Globicephala melas*), Risso’s dolphin (*Grampus griseus*), Harbour porpoise (*Phocoena phocoena*), fetal development, mouth, buccal, oral, pharyngeal, cavity, endoscopy, sectional anatomy, dissection, histology, ontogeny, MRI

## Abstract

**Simple Summary:**

Developmental studies of the dolphin oral cavity have been scarce and were mostly carried out on adult specimens dealing with teeth and lingual development. Moreover, the adult pharyngeal cavity has been mentioned in cetacean monographic encyclopedias and handbooks. In this work, prenatal and perinatal studies of both the oral and pharyngeal cavities were performed on juvenile and adult specimens to better understand these anatomical structures. Our study analyzes these cavities using high-resolution endoscopy to observe changes in the mucosa and to compare these findings with terrestrial mammals. Even though endoscopy was the main technique used, our study was reinforced with Magnetic Resonance Imaging (MRI), anatomical techniques and fetal histology to locate and identify significant structures. Endoscopy of the oral cavity showed some interesting morphological changes. The incisive papilla, teeth, tongue papillae and lateral sublingual recesses and folds were observed in different development stages. The three different parts of the pharynx (oropharynx, laryngopharynx, and nasopharynx) were examined using endoscopy. The histological study helps us to understand the function of the pharyngeal cavity. The nasopharynx contained important structures such as the orifice of the auditory tube and its expansion, the pharyngeal diverticula of the auditory tubes. This special anatomical area was studied using MRI, serial sections and dissections. Some functional considerations are made about both cavities in the five species of odontocetes studied.

**Abstract:**

In this work, the fetal and newborn anatomical structures of the dolphin oropharyngeal cavities were studied. The main technique used was endoscopy, as these cavities are narrow tubular spaces and the oral cavity is difficult to photograph without moving the specimen. The endoscope was used to study the mucosal features of the oral and pharyngeal cavities. Two pharyngeal diverticula of the auditory tubes were discovered on either side of the choanae and larynx. These spaces begin close to the musculotubaric channel of the middle ear, are linked to the pterygopalatine recesses (pterygoid sinus) and they extend to the maxillopalatine fossa. Magnetic Resonance Imaging (MRI), osteological analysis, sectional anatomy, dissections, and histology were also used to better understand the function of the pharyngeal diverticula of the auditory tubes. These data were then compared with the horse’s pharyngeal diverticula of the auditory tubes. The histology revealed that a vascular plexus inside these diverticula could help to expel the air from this space to the nasopharynx. In the oral cavity, teeth remain inside the alveolus and covered by gums. The marginal papillae of the tongue differ in extension depending on the fetal specimen studied. The histology reveals that the incisive papilla is vestigial and contain abundant innervation. No ducts were observed inside lateral sublingual folds in the oral cavity proper and caruncles were not seen in the prefrenular space.

## 1. Introduction

The anatomy of the cephalic region of marine mammals has undergone many evolutionary changes [1]. Amongst all those adaptations in cetaceans, the complete independence of the digestive and respiratory system is one of the most important and has direct implications in the colonization of the aquatic environment. Even though the process of elongation of the skull and the migration of certain flat bones mainly affected the nasal and cranial cavities, the oral cavity also underwent a change in comparison with terrestrial animals, and the repositioning of these bones and the vertical placement of the nasal structures meant that the nasal bones and orifice disappeared from the face.

Inside the cavities surrounded by these bones, the muscles, organs and other anatomical structures also adapted to the new aquatic environment. The tongue, which occupies most of the space within the oral cavity, has the most important role (followed by the teeth) in maintaining the thermoregulatory and feeding [2] functions of terrestrial mammals, but also in acquiring the new function of expelling water from the mouth after feeding, a feature especially seen in Mysticetes.

The oral cavity is not the most studied region in the cetacean head, when compared, for example, with the nasal cavity [3,4,5,6,7,8]. The snout is a sensitive anatomical structure, the first one to have contact with the environment and it performs specific interactions, such as courtship (epicritic tactile sensibility) and defence (protopathic tactile sensibility). There are few articles in the scientific literature about the oral cavity and pharynx, and some of the existing studies may need to be clarified. Most of these articles are about adult specimens, [9,10,11,12], and the few papers on development, both in mysticetes [13,14] and odontocetes [15,16], focus mainly on the tongue of neonates, and do not cover different stages of development, being restricted to only one species [17].

Throughout gestation, bones and other oral structures are in the process of development and their formation continues after birth. Endoscopy is commonly used in dolphin medicine, especially to view the lower respiratory tract (lungs and bronchus) [18,19,20,21,22,23]. It is not often used to visualize the oropharyngeal tract, a region of key importance in stranded dolphins where pathological changes (such as tooth infections or oral obstructions) can be the cause of death.

We include the pharyngeal cavity, as food goes towards the oesophagus and stomach via the laryngopharynx. A comparison with terrestrial mammals will also serve to describe the structures of the head, following the Illustrated Veterinary Anatomical Nomenclature [24] and will aid in our understanding of the function and position of the different structures forming the buccopharyngeal cavity of our small cetaceans.

## 2. Materials and Methods

### 2.1. Animals

In the current study, we analysed the oral complex of 24 odontocetes belonging to five species (*Stenella coeruleoalba*, *Delphinus delphis*, *Globicephala melas*, *Grampus griseus* and *Phocoena phocoena)* of all ages (17 fetuses of different stages, three newborn, two juveniles and two adults) carrying out several diagnostic techniques on all specimens: namely, endoscopy, magnetic resonance imaging (MRI), dissections and histology (Table 1). Additional information is contained in Table S1 (Supplementary Materials). The mothers of each fetus were stranded along the Spanish Atlantic and Mediterranean coasts. The newborn and adult specimens were stranded along the Spanish Mediterranean coast. All stranded specimens were found dead and ethics committee clearance was not necessary. An endoscopic study was carried out, transporting sixteen fetuses and one juvenile to Veterinary Clinic “Bonafé”, La Alberca, Murcia, (Spain) during November 2020. Eight fetuses were transported to the MRI unit to perform scans. One newborn, one juvenile and one adult specimen were used to obtain anatomical sections, the same adult and two fetuses for histological analysis and one newborn for dissection.

### 2.2. Endoscopy

A fixed endoscopy unit (Karl Storz Autocon 200, Tuttlingen, Germany) located at Clínica Veterinaria “Bonafé”, La Alberca (Murcia), Spain with a camera processor (Storz image 1 hub, camera head Karl Storz Image 1 H3 HD, a Storz power led 175) was used to obtain endoscopic images. For the oral, oropharyngeal and laryngopharyngeal cavities, we employed a forward telescope (0° enlarged view, diameter 4 mm, length 14 cm, with incorporated fiber optic light transmission) and for the nasopharyngeal cavity, we used a forward-oblique telescope 30°, diameter 2.7 mm, length 18 cm, with incorporated fiber optic light transmission.

The endoscopic procedure was performed on the fetuses and juvenile specimens following standard protocols. Each specimen was placed in ventral recumbency and facing the endoscopist. Initial image acquisition was of the oral, oropharyngeal and laryngopharyngeal cavities using an optic of 4 mm diameter and 0° vision angle. Following this, the pharyngeal cavity was examined, first visualizing the oropharynx, then turning the optic to obtain a complete exposition of this part of the pharyngeal cavity. After visualizing the epiglottic mucosa, the endoscope was introduced through the left pyriform recess towards the oesophageal vestibule to reach the laryngoesophageal limit and the oesophageal mucosa.

The nasopharyngeal cavity was examined by introducing an optic of 2.7 mm and 30° vision angle protected by a 3 mm sheath forming an irrigation channel, visualizing first the vestibule, then the nasal plugs to the nasal cavity and finally the nasopharynx. All endoscopic images were stored in external and internal hard disks at the CVB (Bonafé Veterinary Clinic) and at the Department of Anatomy and Embryology, Facultad de Veterinaria, Universidad de Murcia, Spain.

### 2.3. Magnetic Resonance Imaging

Magnetic Resonance (MR) images were obtained with a high-field MR apparatus (General Electric Sigma Excite, Schenectady, NA, USA; Centro Veterinario de Diagnóstico por Imagen de Levante, Ciudad Quesada, Alicante, Spain), 1.5 Tesla using a human quadknee coil (dde3, 5, 8, 11,12, gma1) and head coil (dde13-14, grgr1). Images were used to analyze the oral and pharyngeal cavities, and for a special study of the pharyngeal diverticulum of the auditory tube during fetal development. All dolphin specimens were positioned in ventral recumbency. The MR images were transferred to a DICOM workstation. MR images were analyzed with Radiant DICOM viewer. MRI parameters used are in Table S2 (Supplementary Materials). All MR images were stored in external and internal hard disks at the CVDIL (Veterinary Center for Imaging Diagnosis), Ciudad Quesada, Alicante, Spain and the Department of Anatomy and Embryology, Facultad de Veterinaria, Universidad de Murcia, Spain.

### 2.4. Anatomic Evaluation: Sectional Anatomy and Dissection Techniques

One newborn, one juvenile and one adult *Stenella coeruleoalba* were frozen at −20 °C prior to obtaining coronal and sagittal sections of the head. One adult *Stenella coeruleoalba* was frozen at −46 °C prior to obtaining sagittal sections. All specimens were cut with a band saw (Anatomical Lab, Department of Anatomy and Embryology, Universidad de Murcia, Murcia, Spain), obtaining 0.5–0.7 cm thick slices. Head sections and slices were immersed in 10% formaldehyde for preservation and then stored in a cooling chamber (3 °C) at the Department of Anatomy and Embryology, Facultad de Veterinaria, Universidad de Murcia, Spain.

Sixteen fetuses were preserved by immersion in formaldehyde (10%). Two fetuses and one adult were fixed with embalming solution injected into the fetal umbilical artery and vein, and the adult external jugular vein and left auricule. In a *Stenella coeruleoalba* (scomu5), the external jugular vein and the auricles were injected with embalming solution using an electrical pump. Head coronal and sagittal sections and a deep head dissection of one newborn were made to observe the pharyngeal diverticulum of the auditory tube. One newborn specimen was used to study the normal anatomy of the oral and nasal cavities and one fetus and one adult specimen were used to inspect the bony anatomy of the oral and pharyngeal cavities (Table 1).

All specimens used were stored in a cooling and freezer chambers at the Department of Anatomy and Embryology, Facultad de Veterinaria, Universidad de Murcia, Spain.

### 2.5. Histological Analysis

The mucosa of the oral and pharyngeal cavities was histologically analyzed in two fetuses, one *Delphinus delphis* and one adult *Stenella coeruleoalba*. Elongated rectangles of nasal mucosa were removed at different levels of these cavities. Samples were oriented perpendicular to the paraffin block base and then processed using a special saw. Paraffin blocks were cut to obtain slices. Routine histological processing was carried out and sections were stained with Haematoxylin and Eosin. Samples were then photographed with a computed light microscope (Zeiss Axioskop 40, Jena, Germany) with Camera Insight 2 Axiocam 105 color incorporated. Histological sections were stored at the Department of Anatomy and Embryology, Facultad de Veterinaria, Universidad de Murcia, Spain.

## 3. Results

### 3.1. The Oral Cavity

**Figure 1:**
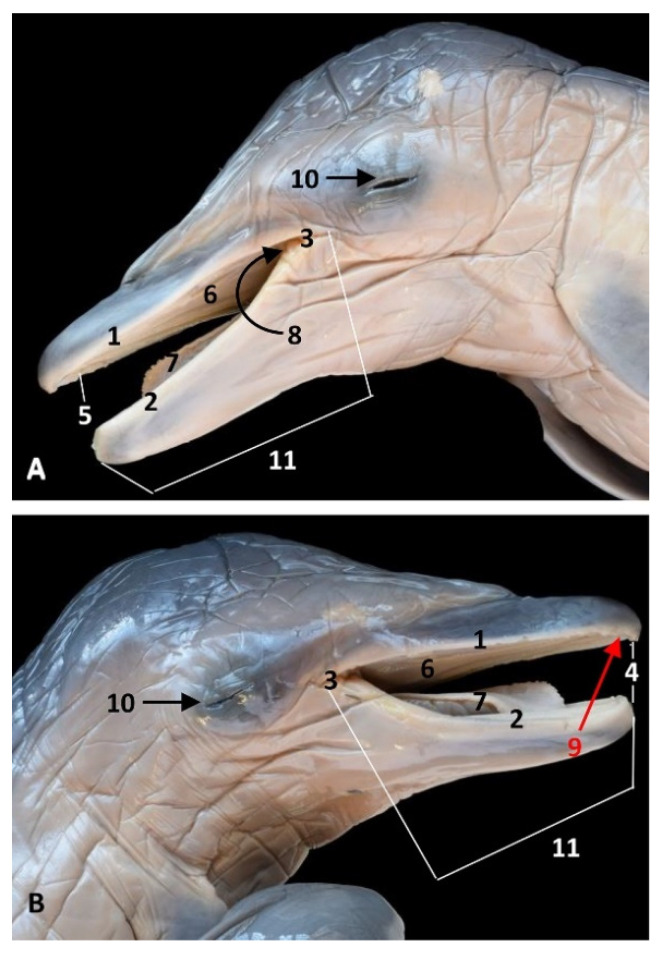
Head of a dolphin fetus showing the oral cavity opened. (**A**) Left aspect; (**B**) Right aspect. 6 months, dde7. 1, Upper lip; 2, Lower lip; 3, Angulus oris; 4, Rima oris; 5, Oral vestibule; 6, Oral cavity roof; 7, Tongue and oral cavity floor: 8, Palatoglossal archs or folds; 9, Incisive teeth (not erupted yet); 10, Eyelids (closed); 11, Oral region.

The oral region is closed during fetal development. We have opened it to view the different parts, starting with the vestibule placed between the lips and the teeth. Beyond the vestibule is the oral cavity proper, which extends from the rostral part of the palatoglossal arch or folds to the lingual aspect of the incisive teeth. The dorsal limit is the oral cavity’s roof, and the ventral limit is defined by the tongue and the proper oral cavity and the prefrenular space (Figure 1).

**Figure 2:**
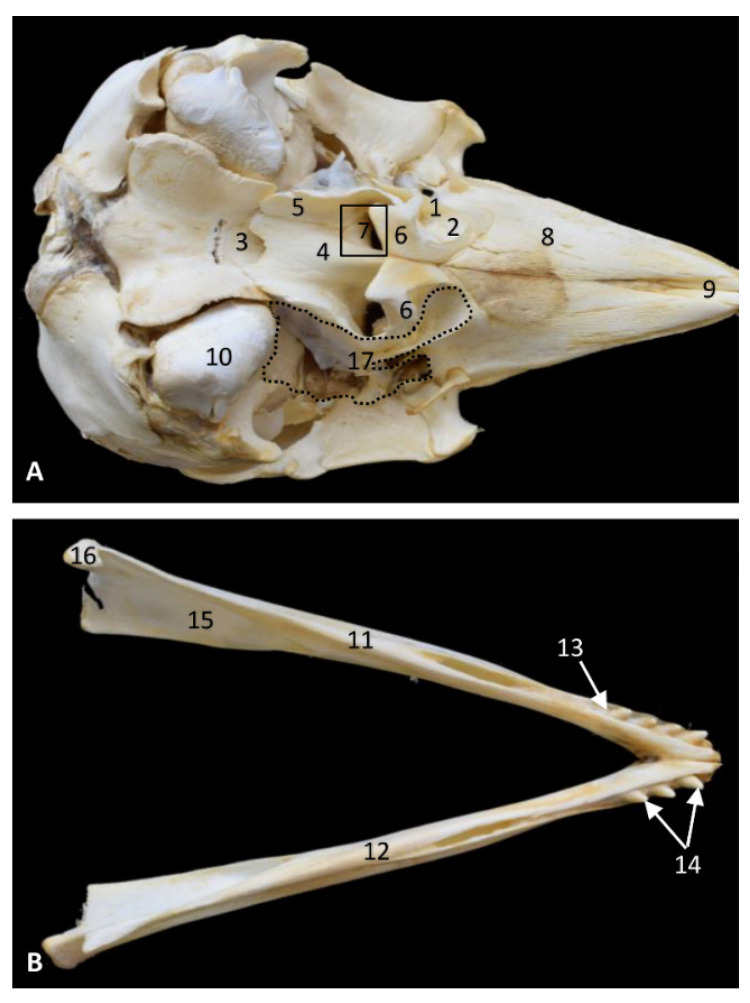
Bony anatomical base of the oral and pharyngeal cavities in a skull. (**A**) Ventral view. Right and left mandibles. (**B**) Dorsal view. Dots show the extension and form of the right pharyngeal diverticulum of the auditory tube. Photography Francisco Gil Cano. 9 months, phop1. 1, Palatine bone: perpendicular lamina; 2, Palatine bone: horizontal lamina; 3, Basisphenoid bone: body; 4, Vomer bone; 5, Pterygoid bone: medial lamina; 6, Pterygoid bone: lateral lamina (incomplete); 7, Choana; 8, Maxillary bone: palatine process; 9, Incisive bone: palatine process; 10, Temporal bone: petrous and tympanic part; 11; Left mandible: body; 12, Right mandible: body; 13, Dental alveolus; 14, Incisive teeth; 15, Mandibular channel; 16, Mandible: condyle; 17, Bony area of pharyngeal diverticulum of the auditory tube.

The bony part of the oral cavity’s roof is composed of the palatine processes of the maxillary and incisive bones as seen in the *Phocoena phocoena* (phog1). The bony ventral limit of the oral cavity is represented by the left and right mandibles. In this specimen, close to birth, teeth have a conic shape. The intermandibular symphysis can be seen. Four incisive teeth were observed (Figure 2).

#### 3.1.1. Endoscopic Study

**Figure 3:**
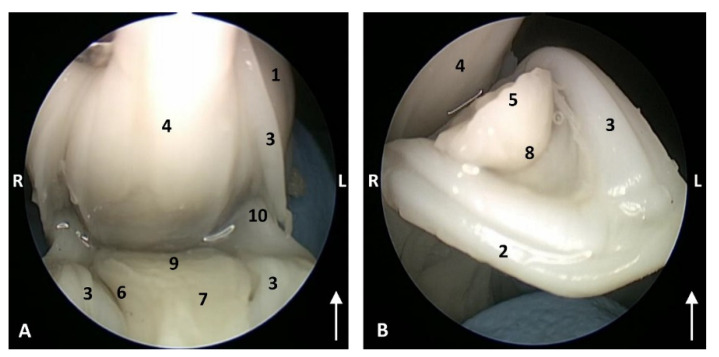
Endoscopic images of the oral cavity. The arrows point to the tip of the mouth. **L** (Left) **R** (Right). (**A**) Oral cavity open. (**B**) Oral cavity proper. 1,5 months, dde1. 1, Upper lip; 2, Lower lip; 3, Gums; 4, Hard palate; 5, Tongue: tip; 6, Tongue: border; 7, Tongue: dorsum; 8, Tongue: ventral part; 9, Tongue: root; 10, Angulus oris.

In the least developed fetus a *Delphinus delphis* (dde1), the lips are proportionally large and immobile. The oral rim, like the blowhole, is tightly closed. A frenulum of the superior and inferior lips is absent. The buccal vestibule is not well-defined at this stage of development. The gums of the mandibles are wider than those of the maxilla. A hard palate is forming. An incisive papilla was not observed. The tongue is short, wide and without papillae (Figure 3).

**Figure 4:**
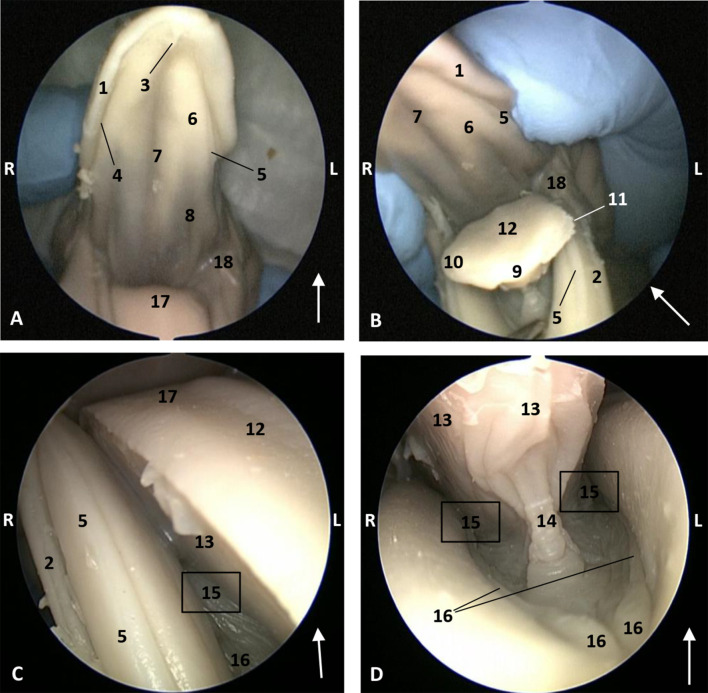
Endoscopic images of the oral cavity. The arrows show the tip of the mouth. **L** (Left) **R** (Right). (**A**,**B**) Oral cavity open. (**C**) Tongue: lateral part. (**D**) Oral cavity proper. 3,5 months, dde2. 1, Upper lip; 2, Lower lip; 3, Incisive papilla; 4, Labial vestibule; 5, Gums; 6, Hard palate; 7, Palatine raphe; 8, Greater palatine groove; 9, Tongue: tip; 10, Tongue: border; 11, Marginal papilla; 12, Tongue: dorsum; 13, Tongue: ventral part; 14, Lingual frenulum; 15, Lateral sublingual recesses; 16, Lateral sublingual folds; 17, Tongue: root; 18, Angulus oris.

In the next fetuses (dde2, dd3), *Delphinus delphis*, a labial vestibule between the gums and lips was observed (not a buccal vestibule). The hard palate shows a middle palatine raphe in all species studied, and only in *Delphinus delphis* are both lateral palatine grooves parallel to the middle hard palate. No transverse ridges of the mucosa of hard palate were observed. We observed the incisive papilla at the tip of hard palate. Marginal papillae extend from the edges to the middle of the tongue. The dorsum of the tongue is smooth with an indistinguishable central groove. In the oral cavity proper, there are two lateral sublingual recesses, with a lateral sublingual fold which thickened rostrally (Figure 4).

**Figure 5:**
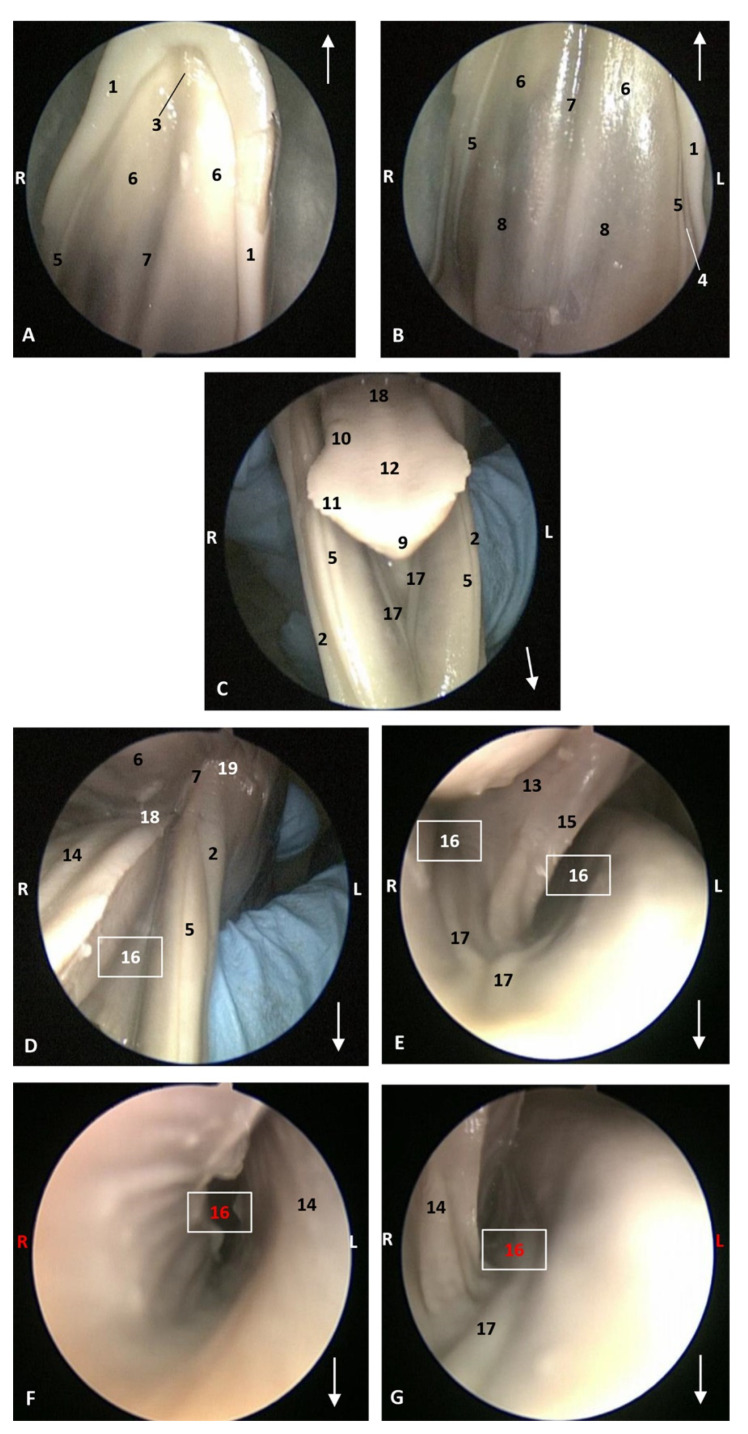
Endoscopic images of the oral cavity. In the different images, the arrows point to the tip of the mouth. **L** (Left) **R** (Right). (**A**,**B**) Hard palate. (**C**) Tongue1. (**D**–**G**) Oral cavity proper. 4 months, dde3. 1, Upper lip; 2, Lower lip; 3, Incisive papilla; 4, Labial vestibule; 5, Gums; 6, Hard palate; 7, Palatine raphe; 8, Greater palatine groove; 9, Tip of the tongue; 10, Tongue: border; 11, Marginal papilla; 12, Tongue: dorsum; 13, Tongue: ventral part; 14, Tongue: longitudinal prominence; 15, Lingual frenulum; 16, Lateral sublingual recess; 17, Lateral sublingual folds; 18, Tongue: root; 19, Angulus oris.

In dde3, the lateral sublingual folds are very thin (Figure 5).

The ventral part of the tongue shows a simple lingual frenulum. In the pre-frenular space no sublingual caruncles were observed. Gums are long and smooth (Figure 4 and Figure 5).

**Figure 6:**
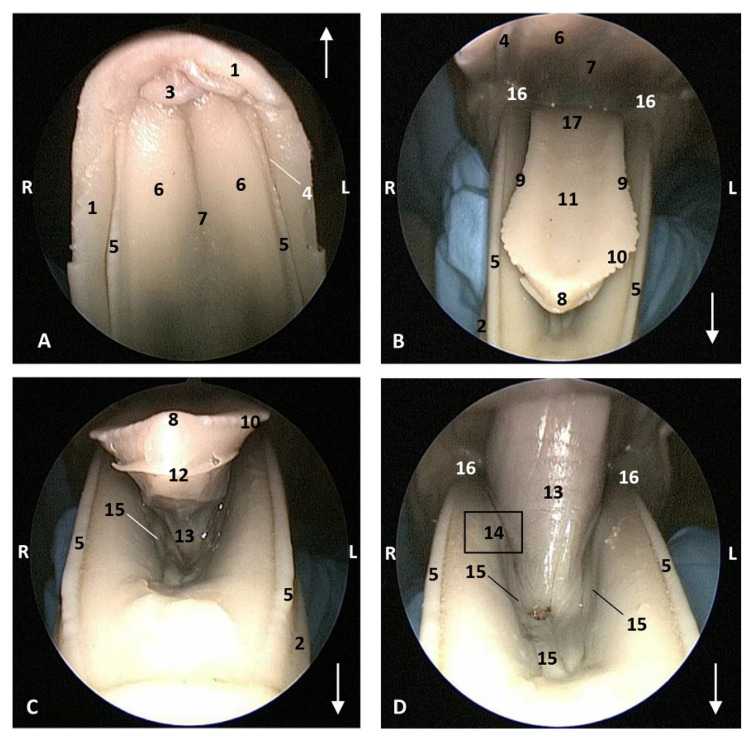
Endoscopic images of the oral cavity. The arrows show the tip of the mouth. **L** (Left) **R** (Right). (**A**) Hard palate. (**B**,**C**) Tongue. (**D**) Oral cavity proper. 4,5 months, scop1. 1, Upper lip; 2, Lower lip; 3, Incisive papilla; 4, Labial vestibule; 5, Gums; 6, Hard palate; 7, Palatine raphe; 8, Tongue: tip; 9, Tongue: border; 10, Marginal papilla; 11, Tongue: dorsum; 12, Tongue: ventral part; 13, Lingual frenulum; 14, Lateral sublingual recess; 15, Lateral sublingual folds; 16, Angulus oris; 17, Tongue: root.

In the *Stenella coeruleoalba* (scop1), one month older than *Delphinus delphis* (dde3), the hard palate shows a palatine raphe and the incisive papilla is a little larger than in *Delphinus delphis* (dde2). The lateral sublingual folds are thin (Figure 6).

**Figure 7:**
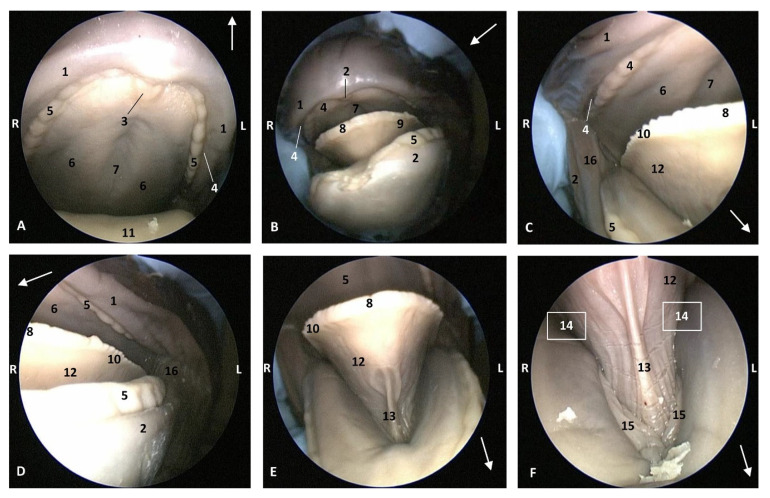
Endoscopic images of the oral cavity. In the different images, the arrow points to the tip of the mouth. **L** (Left) **R** (Right). (**A**,**B**) Oral cavity open. (**C**,**D**) Tongue: lateral part. (**E**,**F**) Oral cavity proper. 5 months, gma1. 1, Upper lip; 2, Lower lip; 3, Incisive papilla; 4, Oral vestibule; 5, Gums covering teeth; 6, Hard palate; 7, Palatine raphe; 8, Tongue: tip; 9, Tongue: border; 10, Marginal papilla; 11, Dorsum of the tongue; 12, Tongue: ventral part; 13, Lingual frenulum; 14, Lateral sublingual recess; 15, Lateral sublingual folds; 16, Angulus oris.

In the *Globicephala melas* (gma1), a little older than *Stenella coeruleoalba* (scop1), the hard palate has a deep palatine raphe and the incisive papilla is larger than in other fetuses. The marginal papillae extend from the tip of the tongue almost to the root. The lingual frenulum is thinner than in other small fetuses. The gums are covering the teeth in both superior and inferior arcades. Teeth are well differentiated in this *Globicephala melas* at this stage (5 months). Also, the sublingual lateral folds are thin but their extremities are starting to thicken (Figure 7).

**Figure 8:**
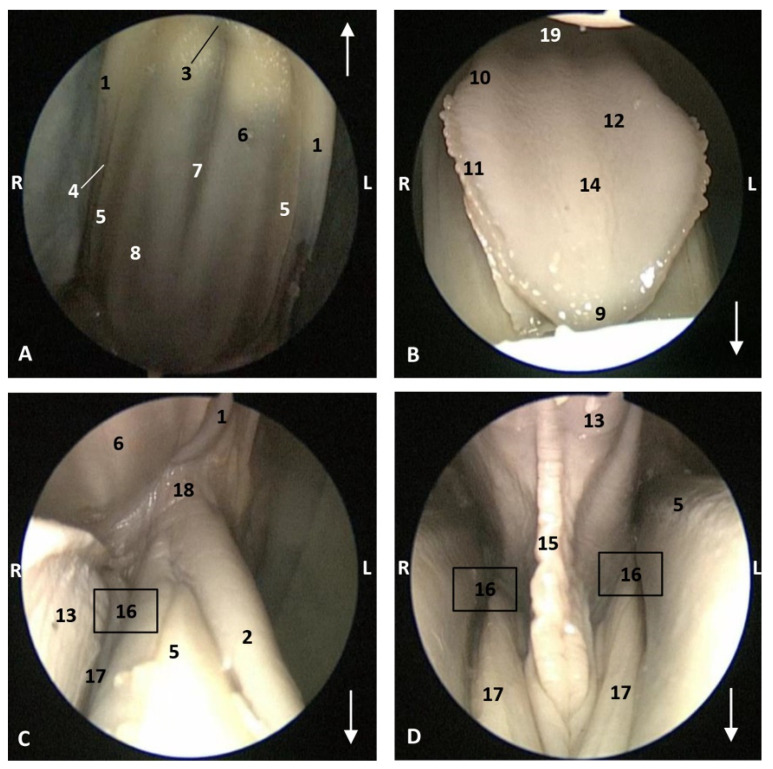
Endoscopic images of the oral cavity. In the different images, the arrow points to the tip of the mouth. **L** (Left) **R** (Right). (**A**) Hard palate. (**B**) Tongue. (**C**,**D**) Oral cavity proper. 6 months, dde8. 1, Upper lip; 2, Lower lip; 3, Incisive papilla; 4, Labial vestibule; 5, Gums; 6, Hard palate; 7, Palatine raphe; 8, Greater palatine groove; 9, Tip of the tongue; 10, Tongue: border; 11, Marginal papilla; 12, Tongue: dorsum; 13, Tongue: ventral part; 14, Tongue: longitudinal prominence; 15, Lingual frenulum; 16, lateral sublingual recesses; 17, Lateral sublingual folds; 18, Angulus oris; 19, Tongue: root.

In a more developed fetus (dde8) *Delphinus delphis,* it was possible to observe marginal papillae extending from the tip to the middle of the tongue, and the lateral sublingual folds were thickening throughout their length (Figure 8).

**Figure 9:**
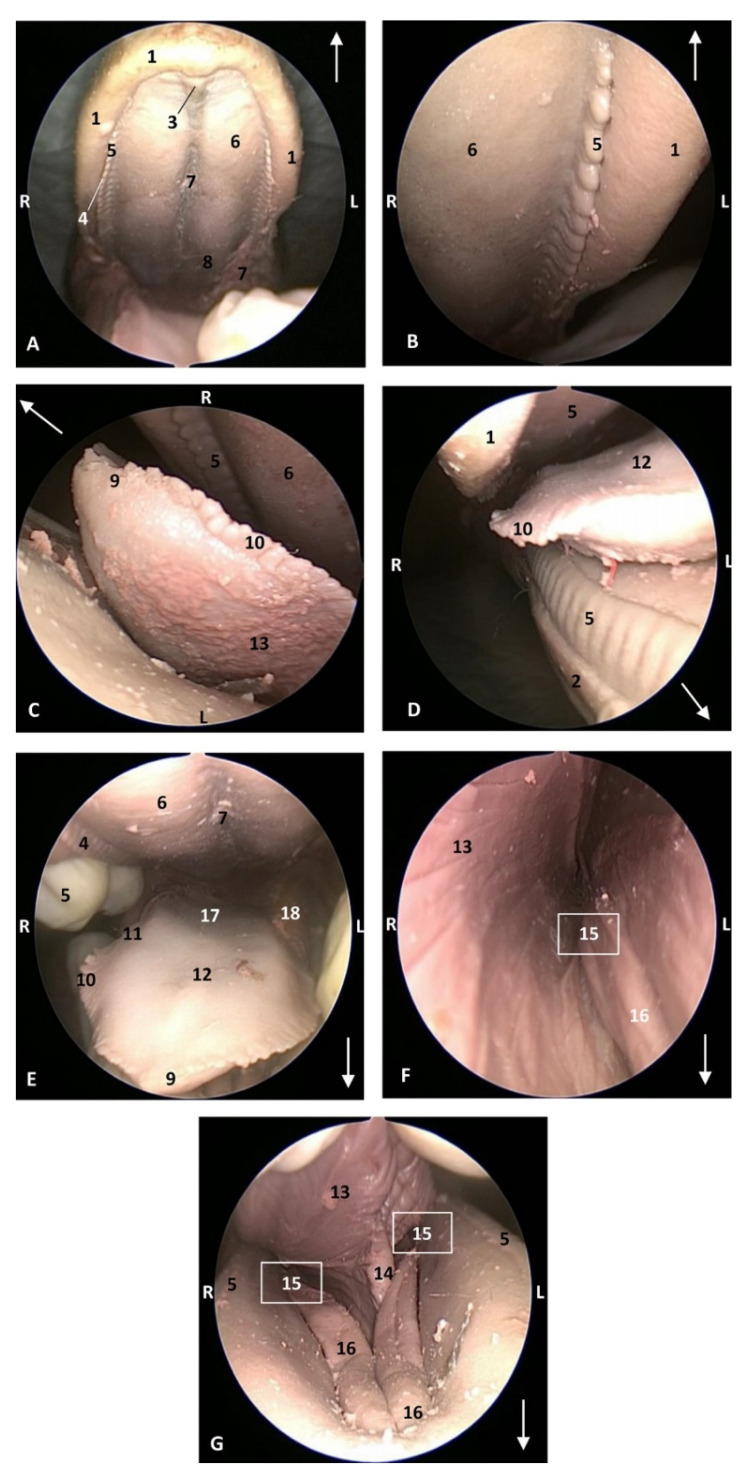
Endoscopic images of the oral cavity. In the different images, the arrow points to the tip of the mouth. **L** (Left) **R** (Right). (**A**) Hard palate. (**B**) Detail of gums. (**C**) Tip of the tongue and prefrenular space. (**D**) Right mandible and lateral part of the tongue. (**E**) Tongue. (**F**) Detail of left lateral sublingual recess. (**G**) Oral cavity proper: prefenular space. 9 months, dde13. 1, Upper lip; 2, Lower lip; 3, Incisive papilla; 4, Labial vestibule; 5, Gums; 6, Hard palate; 7, Palatine raphe; 8, Greater palatine groove; 9, Tongue: tip; 10, Tongue: marginal papilla; 11, Tongue: border; 12, Tongue: dorsum; 13, Tongue: ventral part; 14, Lingual frenulum; 15, lateral sublingual recesses; 16, Lateral sublingual folds; 17, Tongue: root; 18, Angulus oris.

In a *Delphinus delphis* (dde13) close to birth, the teeth of the superior arcade are well developed, unlike those of the inferior arcade. Additionally, the lateral sublingual folds are well dilated (Figure 9).

**Figure 10:**
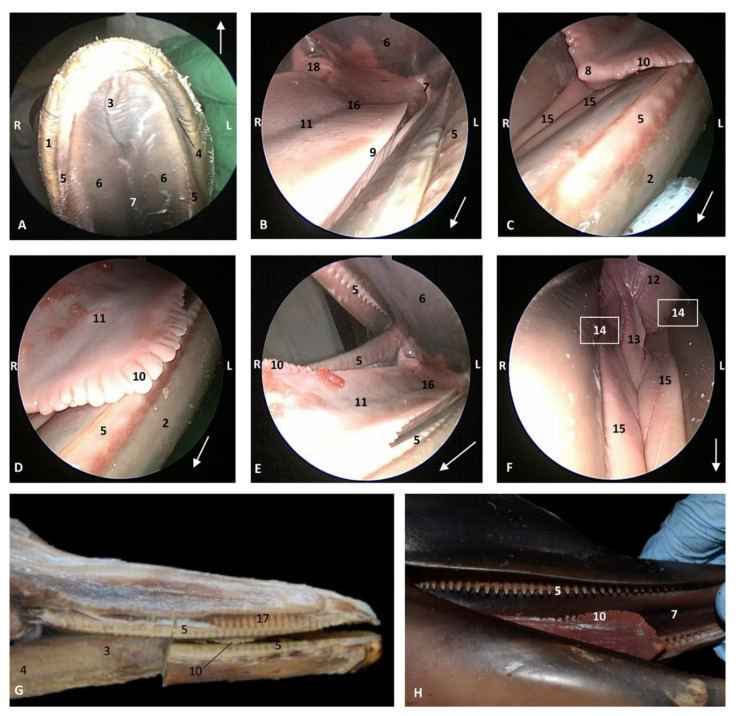
Endoscopic images of the oral cavity. In the different images, the arrow points to the tip of the mouth. **L** (Left) **R** (Right). (**A**) Hard palate. (**B**) Tongue. (**C**–**F**) Tongue, oral cavity proper and prefenular space. newborn, scumu2. (**G**) Deep dissection of the newborn dolphin head after removing skin and partial section of the right mandible. Observe that rostral teeth during lactation period do not erupt to protect mother’s nipple. newborn, scoce1. (**H**) Detailed image of the marginal papilla and teeth without gum covering the clinic crown. A palatal raphe in hard palate is present. newborn, scomu1. 1, Upper lip; 2, Lower lip; 3, Incisive papilla; 4, Labial vestibule; 5, Gums; 6, Hard palate; 7, Palatine raphe; 8, Tongue: tip; 9, Tongue: border; 10, Tongue: marginal papilla; 11, Tongue: dorsum (middle tongue groove); 12, Tongue: ventral part; 13, Lingual frenulum; 14, lateral sublingual recess; 15, Lateral sublingual folds; 16, Tongue: root; 17, Teeth roots; 18, Angulus oris.

In the youngest newborn *Stenella coeruleoalba* (scoce1), all teeth are covered by gums. Teeth eruption can be seen in the two older newborn specimens (scomu1 and scomu2) where the caudal teeth start to erupt, but the rostral teeth are covered by gums and the incisive teeth are not well developed. Also, the marginal papillae are well developed and decrease progressively towards the root. (Figure 10).

#### 3.1.2. MRI Study

**Figure 11:**
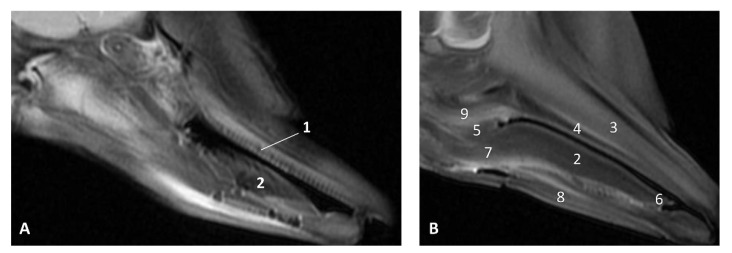
Images of the oral cavity. MR sagittal images is oriented so that the rostral is to the right. (**A**) T2 FrFSE sagittal plane. 6 months, dde8. Image of the oral cavity. (**B**) T2 FrFSE sagittal plane. Quadknee coil. 8 months, dde11. 1, Teeth (under gum); 2, Tongue: body; 3, Maxillary bone; 4, Hard palate; 5, Soft palate; 6, Tongue: apex; 7, Tongue: root; 8, Mandibles; 9, Pterygoid and palatine bones.

The T2 MRI image of the oral cavity in this *Delphinus delphis* (dde11) shows that the maxillary bone is medium hyperintense with respect to the hypointense hard palate. The superficial mucosa of tongue is slightly hyperintense with respect to the hypointense depressor, protractor and retractor muscles of the tongue. A hyperintense stratum under the tongue muscles is probably due to the high rate of irrigation of these muscles. We observed that non-erupted teeth can be seen under the gums (Figure 11).

#### 3.1.3. Histological Study

**Figure 12:**
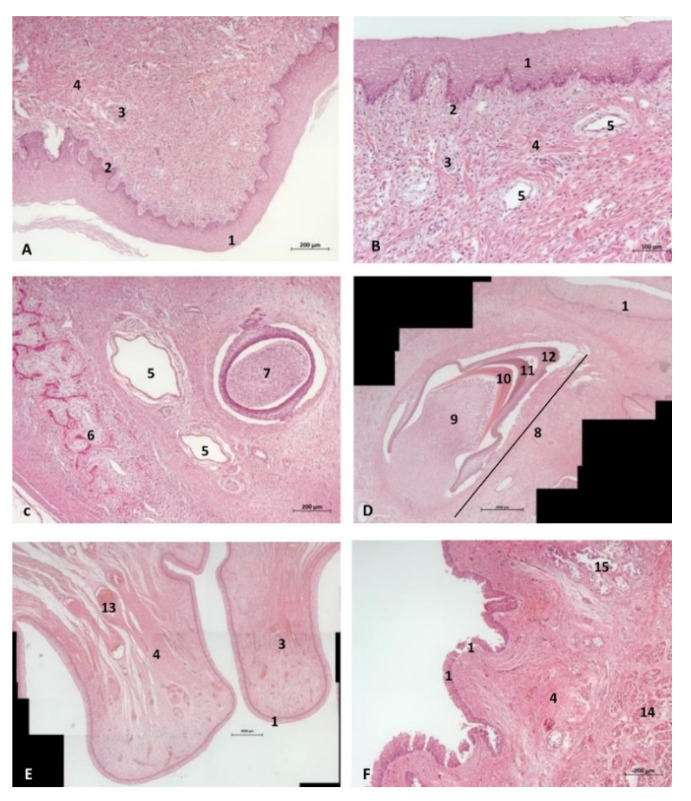
(**A**,**B**) Histological study of the oral cavity. (**A**–**C**) Hard palate: incisive papilla. (**D**) Tooth. (**E**) Oral cavity proper: sublingual lateral fold. (**F**) Tongue: root. 10 months, dde14. 1, Epidermis; 2, Papillary stratum; 3, Nervous tissue; 4, Connective tissue; 5, Lymphatic vessels; 6, Bony tissue; 7, Dental structure initial development transverse sectioned; 8, Dental structure development sagittal sectioned; 9, Dental papilla; 10, Dentin; 11, Enamel; 12, Cementum; 13, Venous vessels; 14, Striated muscle; 15, Mucous glands.

**Figure 13:**
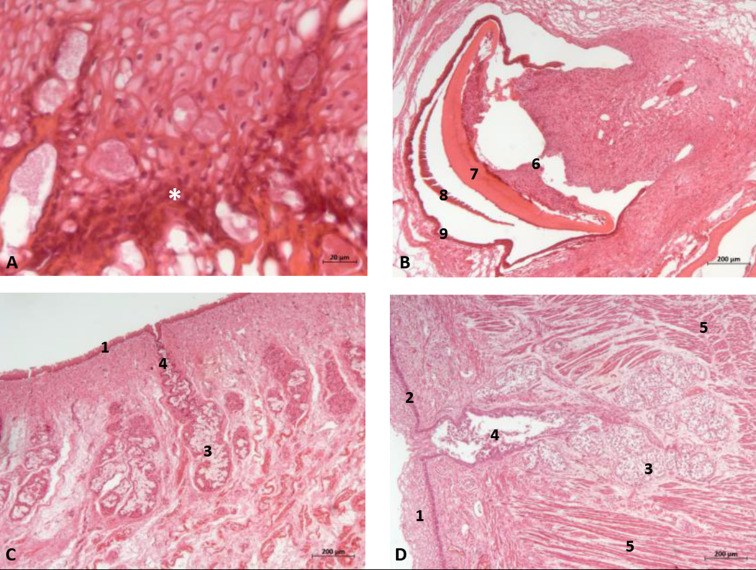
(**A**,**B**) Histological study of the oral cavity. (**A**) Sublingual lateral fold: pigmented epithelium in basal stratum (*). (**B**) Tooth. (**C**,**D**) Tongue: root. 7.5 months, dde10. 1, Epidermis; 2, Papillary stratum; 3, Mucous glands; 4, Secretor duct; 5, Striated muscle: proper muscle tongue; 6, Dental papilla 7, Dentin; 8, Enamel; 9, Cementum.

Vestigial incisive papillae appear with a keratinized pseudostratified epithelium. Conduits arriving at papilla were not seen, nor was the vomeronasal organ *Delphinus delphis* fetus (dde14). Abundant lymphatic vessels were observed in the dermis. Developing teeth were observed covered by gum tissue. The histological structure of teeth shows the inner dentin, covered by enamel and all parts (crown, neck, and root) covered by cementum. The sublingual lateral folds have a mucosa with abundant vessels and mucous glands while neither the sublingual caruncule nor the orobasal organ were observed (Figure 12 and Figure 13).

The sublingual lateral fold showed a pigmented epithelium at its basal stratum. The tongue shows a striated muscle base and abundant sub-epithelial mucous glands are visible, along with their secretory ducts (Figure 13).

**Figure 14:**
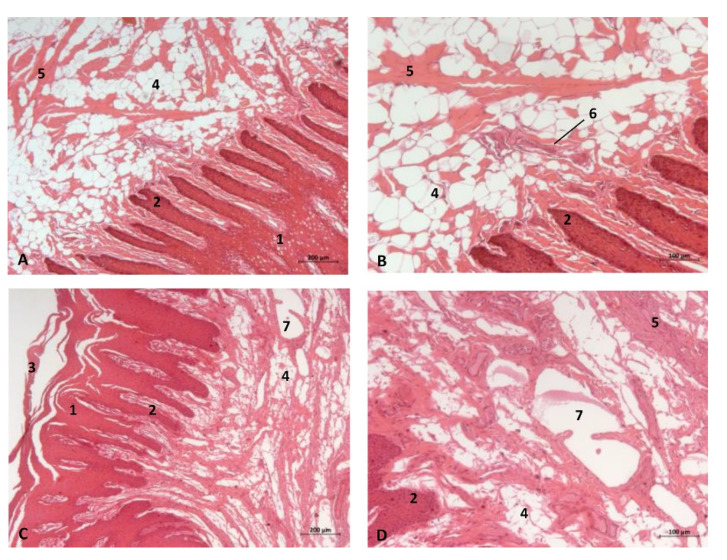
(**A**,**B**) Histological study of the oral cavity. (**A**,**B**) Hard palate: incisive papilla. (**C**,**D**) Oral cavity proper: sublingual lateral fold. Adult, scomu6. 1, Epidermis; 2, Papillary stratum; 3, Corneum stratum; 4, Fat tissue; 5, Connective tissue; 6, Remains of epithelial duct; 7, Lymphatic vessels.

In an adult *Stenella coeruleoalba* (scomu6), the incisive papilla showed a well-developed papillary stratum. The dermis contains abundant fat tissue and nests of epithelial ducts in regression (Figure 14A,B). The lateral sublingual recess has a keratinized epithelium with mucous glands and a well-developed papillary stratum (Figure 14).

### 3.2. The Pharyngeal Cavity

**Figure 15:**
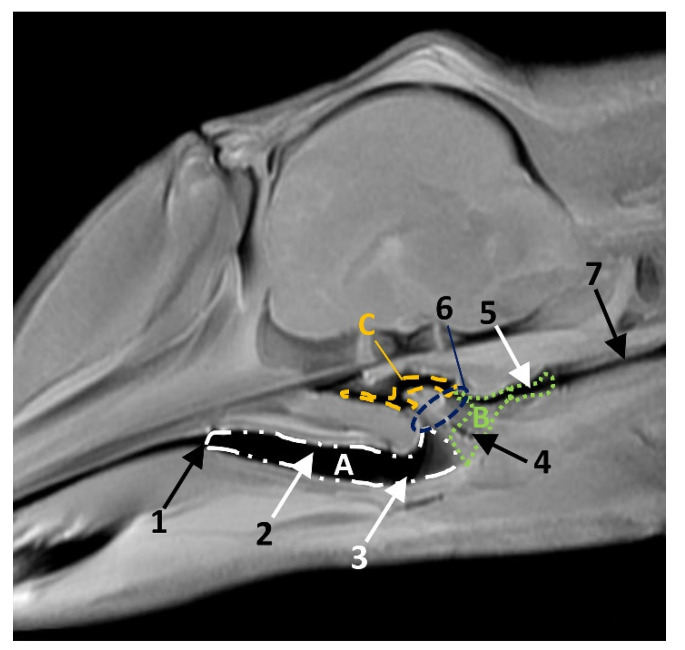
Head of a dolphin fetus, showing the pharyngeal cavity. (**A**) Oropharynx; (**B**) Laryngopharynx; (**C**) Nasopharynx. MRI sagittal T1 SE sequence. 10 months, dde14. 1, Isthmus of the fauces; 2, Fauces; 3, Epiglottic vallecula; 4, Piriform recess; 5, Oesophageal vestibule; 6, Intrapharyngeal orifice: 7, Oesophagus mucosa.

The pharynx is a musculo-membranous cavity divided into three parts: the oropharynx linking the oral cavity with the oesophagus, the nasopharynx connecting the nasal cavity with the larynx, and the laryngopharynx, which is an intermediate cavity caudal to the oropharynx and caudoventral to the nasopharynx. The laryngopharynx connects the oral cavity to the stomach and allows the aditus laryngis enter to the nasopharyngeal cavity crossing, only in cetaceans (Figure 15), the intrapharyngeal orifice.

The bony roof of oropharynx is composed of the palatine bone and the pterygoid bone laminae (lateral and medial). Additionally, the nasopharynx is delimited dorsally by the vomer wings and the medial lamina of the pterygoid bone (Figure 2). Ventrally, the hyoid apparatus attaches to the root of the tongue and the larynx to the base of the cranium.

#### 3.2.1. Study of Oropharynx, Nasopharynx and Laryngopharynx

The oropharynx begins at the isthmus of the fauces, continues with a conduit (fauces) and finishes at the lingual aspect of the epiglottic cartilage (Figure 15).

##### Endoscopic Study

**Figure 16:**
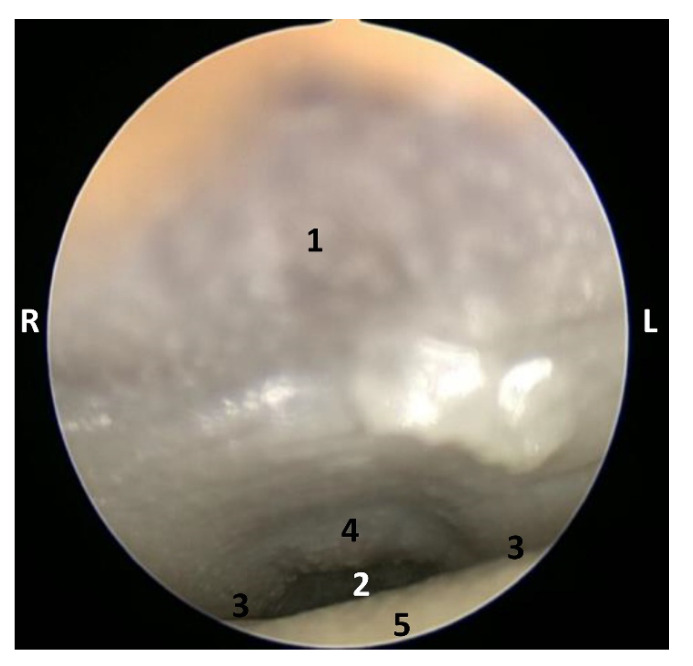
Endoscopic image of the pharyngeal cavity: oropharynx. **L** (Left) **R** (Right). Fauces: isthmus. 3.5 months, dde2. 1, Hard palate; 2, Isthmus of the fauces; 3, Arcus palatoglossus or palatoglossus folds; 4, Soft palate; 5, Tongue: root.

The endoscopic study began at the **oropharynx**, showed a tightly closed isthmus of the fauces in a young fetus, a *Delphinus delphis* (dde2). The endoscope could not cross this gate (Figure 16).

**Figure 17:**
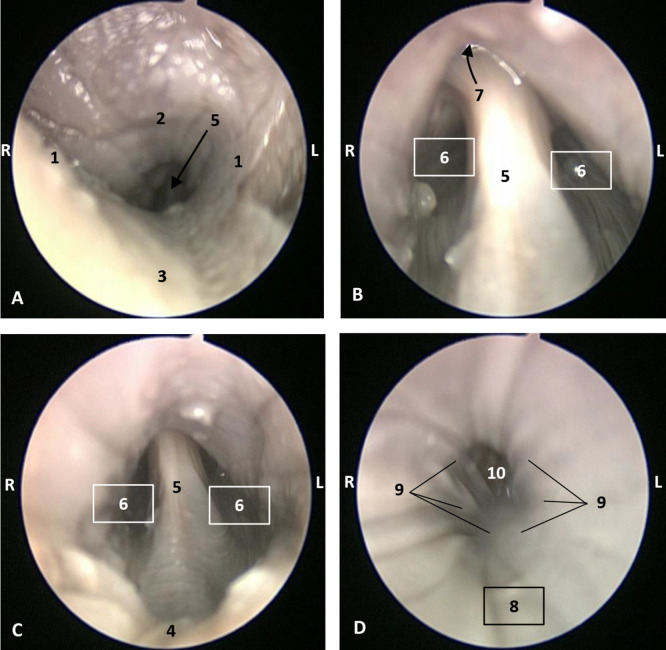
Endoscopic image of the pharyngeal cavity. **L** (Left) **R** (Right). (**A**–**C**) Oropharynx, (**A**) Fauces. (**B**–**D**) Laryngopharynx. 4 months, dde3. 1, Arcus palatoglossus or palatoglossus folds; 2, Soft palate; 3, Tongue: root; 4, Epiglottic vallecula; 5, Epiglottis: lingual surface (mucosa); 6, Piriform recess; 7, Intrapharyngeal orifice (nasopharynx); 8, Oesophageal vestibule; 9, Pharyngoesophageal limit; 10, Oesophageal mucosa.

The endoscope was passed into the fauces in an older fetus, a *Delphinus delphis* (dde3) showing a bright mucosa. No lymphoreticular tissue in the floor (tongue), walls (palatoglossus archs or folds) or roof (soft palate) of the fauces was observed. The soft palate is inserted into the ventral crest formed between the lateral and medial lamina of the pterygoid bones. The palatoglossal archs or folds connect to the soft palate through the tongue root. At the end of fauces a soft vallecula continues dorsally with the lingual aspect of the mucosa of epiglottic cartilage. At this level we dorsally observed the intrapharyngeal orifice to allow entry of the larynx into the **nasopharynx**. Additionally, the **laryngopharynx** begins with a piriform recess on either side of the larynx cartilages. The left piriform recess is wider than the right one. The dilated oesophageal vestibule is caudal to the recesses whose mucosa is arranged in longitudinal folds changing to small quadrangular folds where the oesophageal mucosa begins (Figure 17).

**Figure 18:**
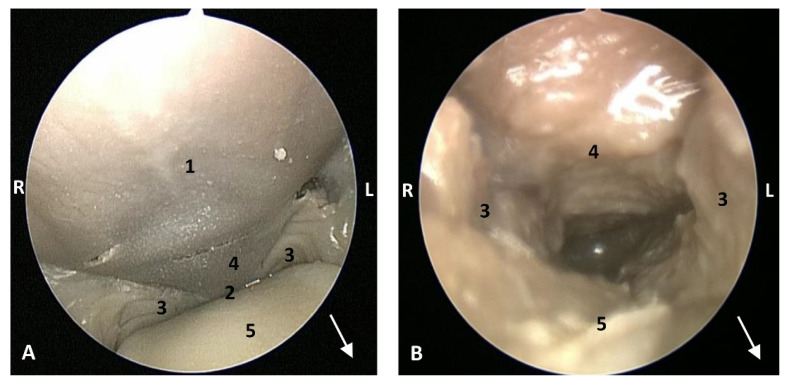
Endoscopic image of the pharyngeal cavity: oropharynx. The arrows show where is the tip of the mouth. **L** (Left) **R** (Right). (**A**) Fauces: isthmus. (**B**) Fauces: inside. 4.5 months, scop1. 1, Hard palate; 2, Isthmus of the fauces (closed); 3, Arcus palatoglossus or palatoglossus folds; 4, Soft palate; 5, Tongue: root.

**Figure 19:**
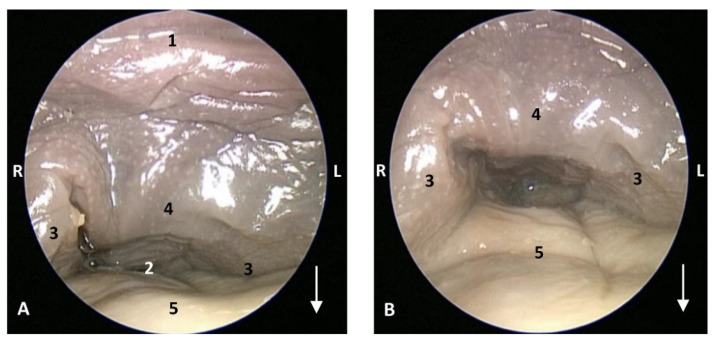
Endoscopic image of the pharyngeal cavity: oropharynx. The arrows show where is the tip of the mouth. **L** (Left) **R** (Right). (**A**) Fauces: isthmus. (**B**) Fauces: inside. 5 months, gma1. 1, Hard palate; 2, Isthmus of the fauces (closed); 3, Arcus palatoglossus or palatoglossus folds; 4, Soft palate; 5, Tongue: root.

Both the *Stenella coeruleoalba* (scop1) and *Globicephala melas* (gma1) fetuses had a well-developed mucosa at the isthmus and only a narrow passage to the fauces which had a pale lingual mucosa and a grey/brown colour in its walls and roof (Figure 18 and Figure 19).

**Figure 20:**
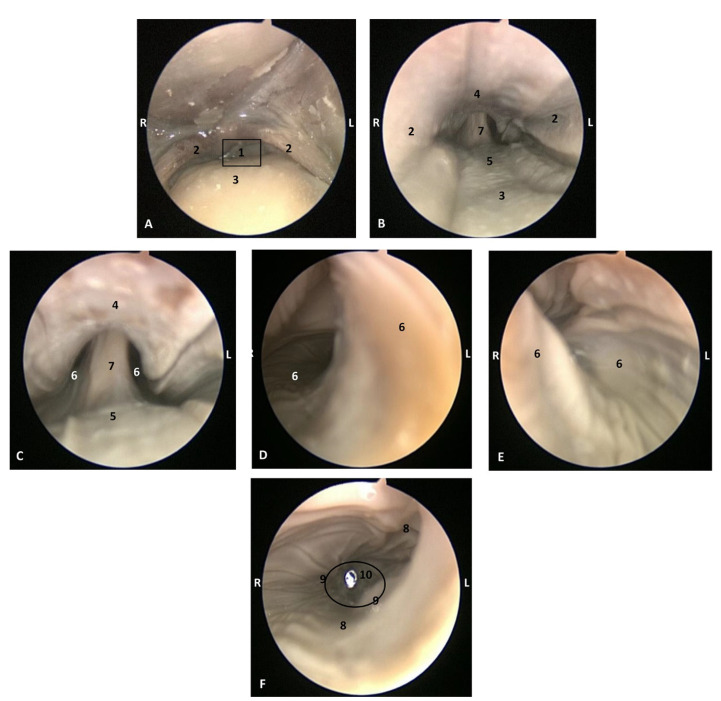
Endoscopic images of the pharyngeal cavity. **L** (Left) **R** (Right). (**A**–**C**) oropharynx. (**B**) Fauces. (**C**–**F**) Laryngopharynx. 6 months, dde8. 1, Isthmus of the fauces; 2, Arcus palatoglossus; 3, Tongue: root; 4, Soft palate; 5, Epiglottic vallecula; 6, Piriform recess; 7, Epiglottis: mucosa. 8, Oesophageal vestibule; 9, Pharyngoesophageal limit; 10, Oesophageal mucosa.

A well-defined fauces was observed in an older *Delphinus delphis* (dde8) and also a broad left piriform recess, with longitudinal folds finishing at the oesophageal vestibule (Figure 20).

**Figure 21:**
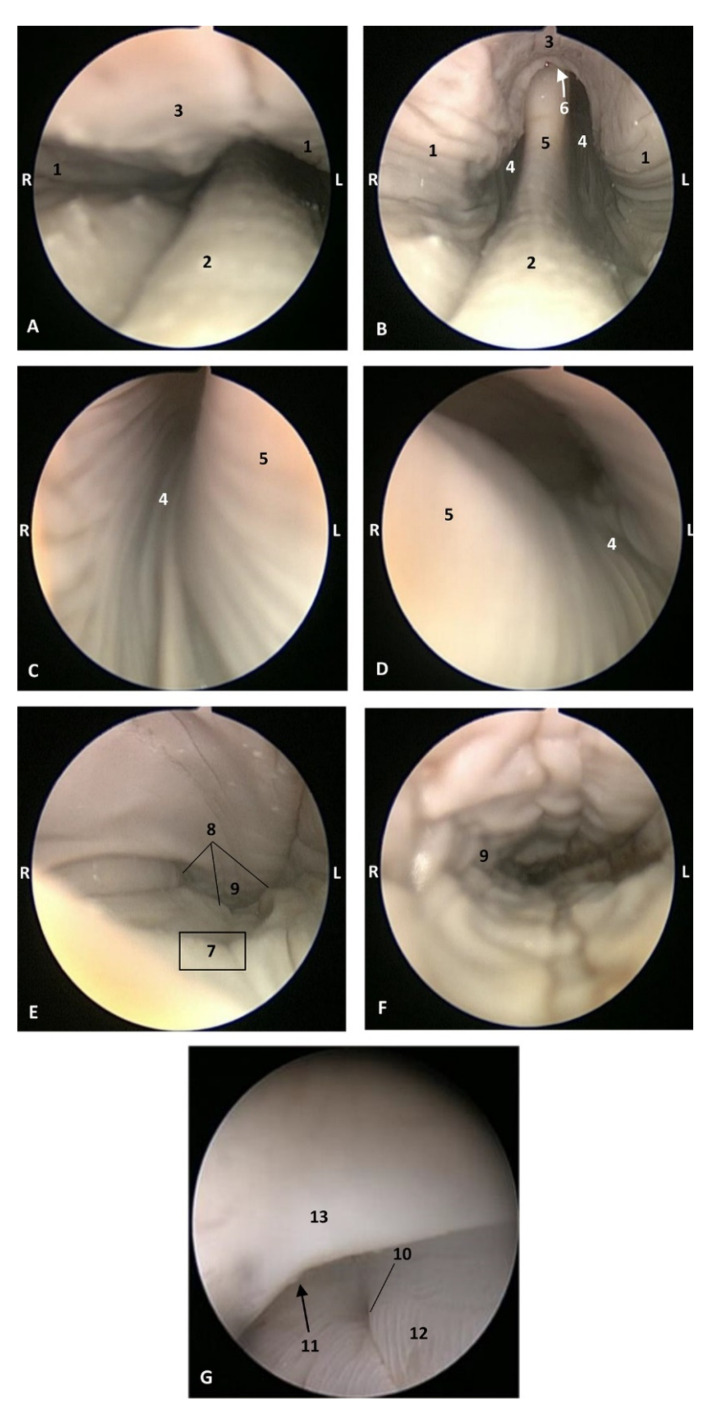
Endoscopic images of the pharyngeal cavity. **L** (Left) **R** (Right). (**A**,**B**) oropharynx: fauces. (**B**–**F**) Laryngopharynx. (**G**) Left nasopharynx. 7 months, dde9. 1, Arcus palatoglossus; 2, Tongue: root; 3, Soft palate; 4, Piriform recesses (laryngopharynx); 5, Epiglottis: mucosa. 6, Intrapharyngeal orifice (entrance to nasopharynx); 7, Oesophageal vestibule; 8, Pharyngoesophageal limit; 9, Oesophageal mucosa; 10, Pharyngeal orifice of the auditory tube; 11, Choanae; 12, Nasopharyngeal mucosa: longitudinal or striated folds; 13, Nasal septum: vomer bone.

In this well-developed fetus (*Delphinus delphis*) (dde9), the endoscope could pass into the choanae to see the nasopharynx and the pharyngeal orifice of the auditory tube; alsothe longitudinal folds changing to small quadrangular folds where the oesophageal mucosa begins (Figure 21). The oropharyngeal mucosa is thickening, the longitudinal folds in the piriform recesses of the laryngopharynx are thin, and a clear difference between the mucosa of the oesophageal vestibule and oesophagus was seen.

**Figure 22:**
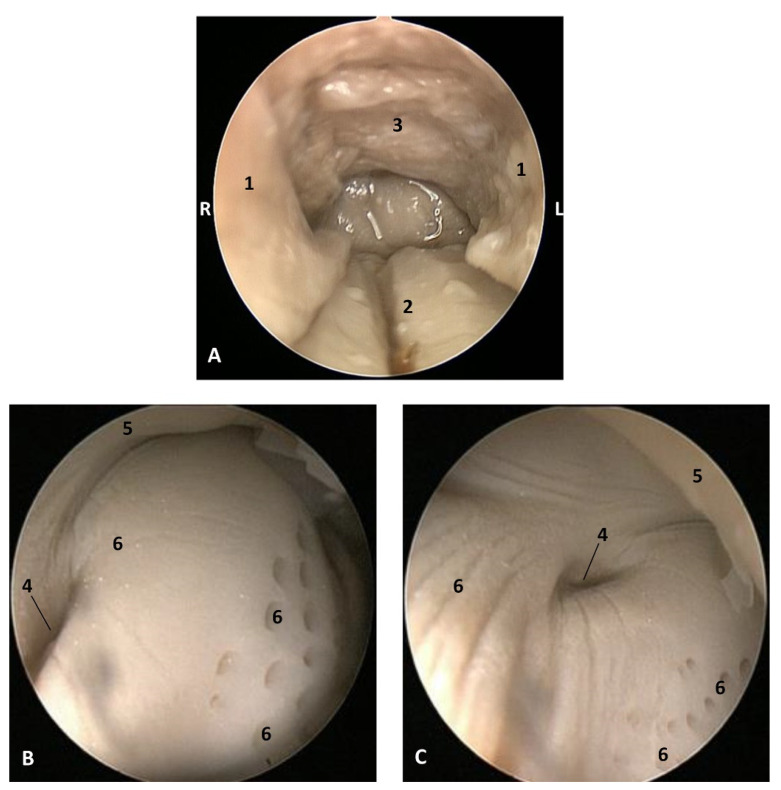
Endoscopic images of the pharyngeal cavity. **L** (Left) **R** (Right). (**A**) Oropharynx: fauces. (**B**) Left nasopharynx. (**C**) Right nasopharynx. 8 months, dde11. 1, Arcus palatoglossus or palatopharyngeal folds; 2, Tongue: root; 3, Soft palate; 4, Pharyngeal orifice of the auditory tube; 5, Nasal septum: vomer bone; 6, Nasopharyngeal mucosa: longitudinal or striated folds and small openings.

The mucosa of the fauces continues to thicken and has a bright aspect in a *Delphinus delphis* fetus (dde11). Additionally, in the nasopharynx, the mucosa shows longitudinal folds and small openings surrounding the pharyngeal orifice of the auditory tube (Figure 22).

**Figure 23:**
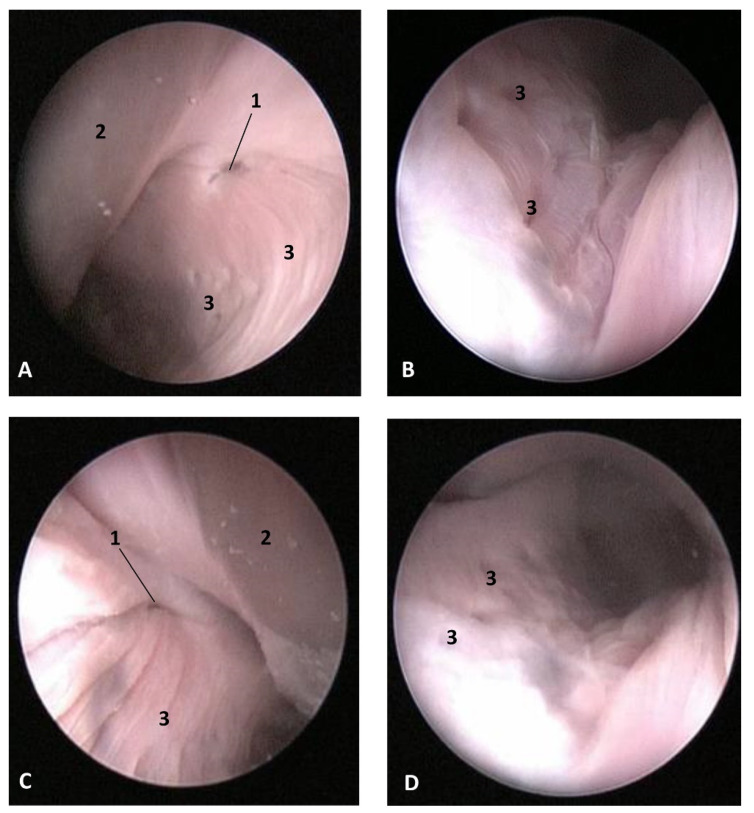
Endoscopic images of the pharyngeal cavity. **L** (Left) **R** (Right). (**A**,**B**) Left nasopharynx. (**C**,**D**) Right nasopharynx. Juvenile, scomu4. 1, Pharyngeal orifice of the auditory tube; 2, Nasal septum: vomer bone; 3, Nasopharyngeal mucosa: longitudinal or striated folds with small holes.

In a juvenile dolphin, we could observe the pinkish mucosa of the nasopharynx with longitudinal folds, but the small holes had less border definition (Figure 23).

##### Histological Study

**Figure 24:**
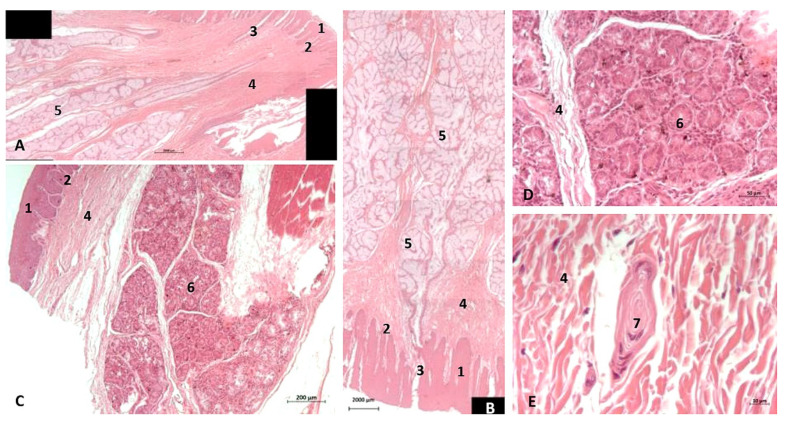
(**A**,**B**) Histological study of the pharyngeal cavity. (**A**) Fauces: soft palate. (**B**) Tongue: root. (**C**) Pharynx: mucosa (**D**,**E**). Detail of pharyngeal mucosa. Adult, scomu6. 1, Epidermis; 2, Papillary stratum; 3, Secretor ducts; 4, Connective tissue; 5, Deep mucous glands; 6, Deep serosa glands; 7, Vater-Paccini corpuscle.

The histological results show that the epidermis of the oropharynx at the soft palate level has a tightly papillary stratum with deep mucous glands and abundant mucous glands in its submucosa. Additionally, at the level of the isthmus of the fauces, histology shows a connective tissue stratum deep to the epidermis, containing many deep mucous glands (Figure 24A,B). The nasopharynx shows a respiratory mucosa with an anfractuous papillary stratum, below which is a wide connective stratum, and finally a deep serous gland close to striated muscle. Additionally, we have located Vater–Paccini corpuscles near the auditory duct between striated muscles (Figure 24C–E). No lymphoreticular tissue was observed.

##### MRI Study

**Figure 25:**
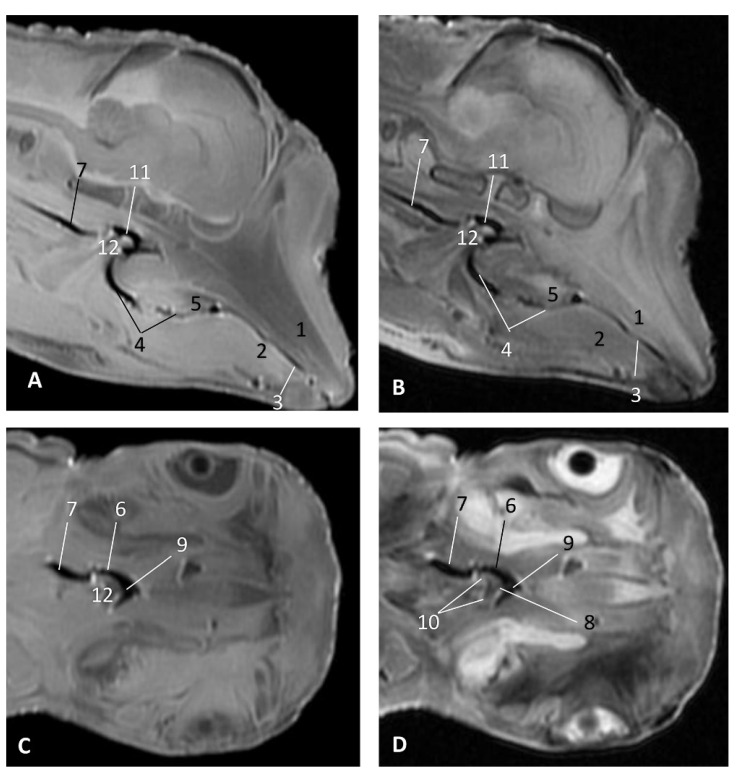
Images of the oral and pharyngeal cavity. MR sagittal and coronal images are oriented so that the rostral is to the right. (**A**) T1 SE sagittal, (**B**) T2 FrFSE sagittal, (**C**) T1 SE coronal and (**D**) T2 FrFSE coronal planes. 5 months, gma1. 1, Hard palate; 2, Tongue; 3, Oral cavity (closed); 4, Oropharynx: fauces; 5, Oropharynx: soft palate; 6, Laryngopharynx: left piriform recess; 7, Laryngopharynx: oesophageal vestibule; 8, Epiglottis cartilage; 9, Epiglottic vallecula; 10, Arytenoid cartilages; 11, Nasopharynx; 12, Larynx.

The MRI sagittal images show a pharyngeal cavity in a *Globicephala melas* fetus (gma1) and we could appreciate the oropharynx (fauces), the **nasopharynx** and the oesophageal vestibule hypointense in both T1 and T2 sequences(Figure 25A,B). Coronal T1 and T2 sequences show the piriform recess alongside the larynx (Figure 25C,D).

#### 3.2.2. Special Study of Nasopharynx and Pharyngeal Diverticulum of the Auditory Tube (PDAT)

(a) MRI study

**Figure 26:**
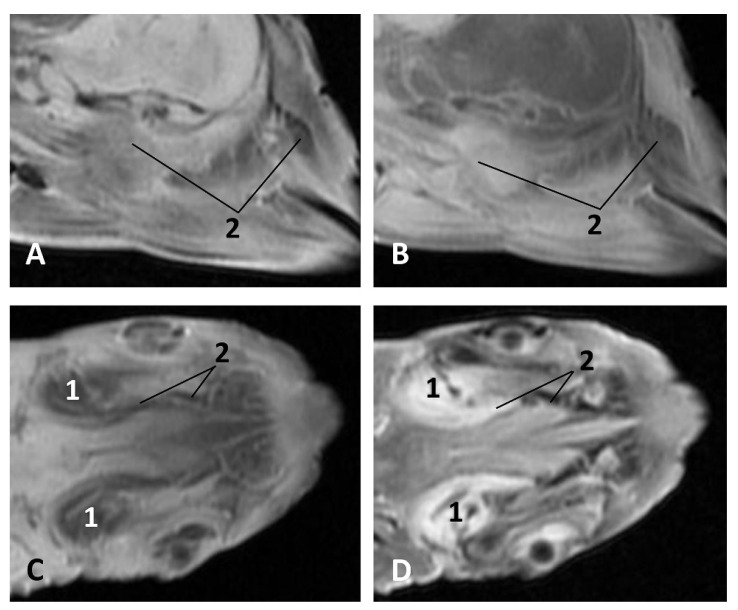
Images of the pharyngeal cavity. MR sagittal and coronal images are oriented so that the rostral is to the right. (**A**) T1 SE sagittal, (**B**) T2 FrFSE sagittal, (**C**) T1 SE coronal and (**D**) T2 FrFSE coronal planes. 4 months, dde3. 1, Inner and middle ear; 2, Pharyngeal diverticulum of the auditory tube.

In MRI, we can appreciate, in early fetal stages, a bilateral structure within the laryngopharyngeal cavity, each named as a *pharyngeal diverticulum of the auditory tube* (PDAT). These are connected through the musculotubaric channel with the middle ear (temporal bone: petrous and tympanic part). In a young *Delphinus delphis* fetus (dde3), it appears in sagittal sections as a hyper/hypointense area seen caudal and rostrally, respectively (Figure 26A,B), and also in coronal sections (Figure 26C,D).

**Figure 27:**
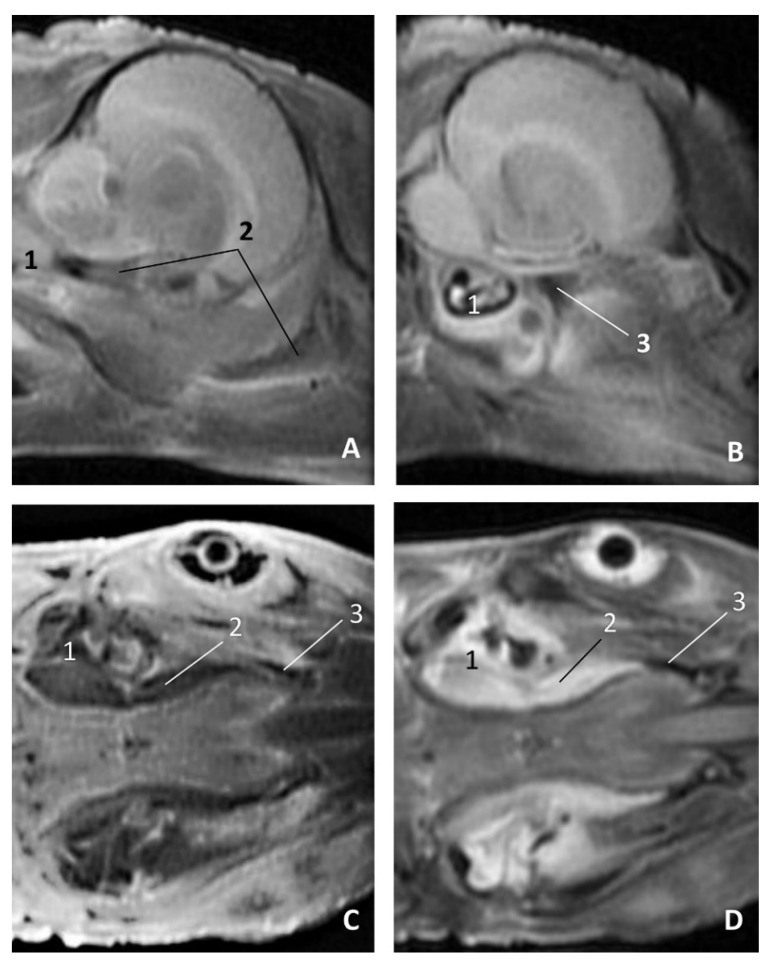
Images of the pharyngeal cavity. MR coronal and sagittal images are oriented so that the rostral is to the right. (**A**,**B**) T2 FrFSE sagittal, (**C**) T1 SE and (**D**) T2 FrFSE coronal planes. 5.5 months, dde5. 1, Inner ear; 2, Pharyngeal diverticulum of the auditory tube: moderate hyperintense area (vascular); 3, Pharyngeal diverticulum of the auditory tube: hypointense area (air).

**Figure 28:**
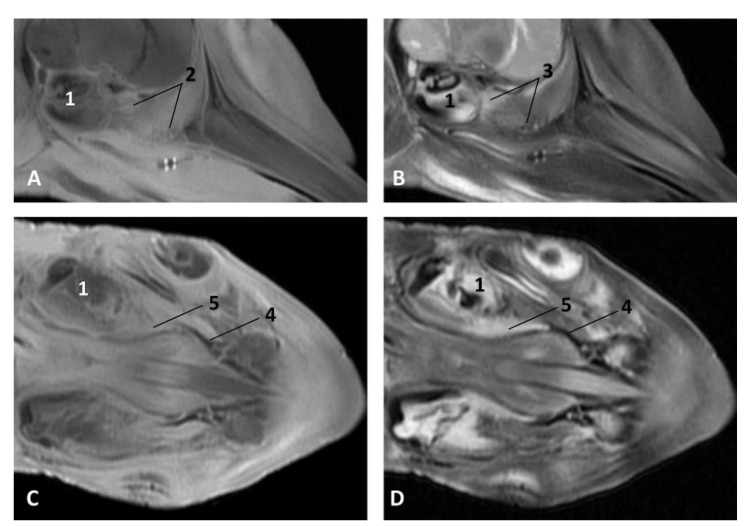
Images of the pharyngeal cavity. MR sagittal and coronal images are oriented so that the rostral is to the right. (**A**) T1 SE sagittal, (**B**) T2 FrFSE sagittal, (**C**) T1 SE coronal and (**D**) T2 FrFSE coronal planes. 6 months, dde8. 1, Inner ear; 2, Pharyngeal diverticulum of the auditory tube: moderate hyperintense area (vascular); 3, Pharyngeal diverticulum of the auditory tube: moderate hypointense area; 4, Pharyngeal diverticulum of the auditory tube: hypointense area (air); 5, Pharyngeal diverticulum of the auditory tube: hyperintense area (vascular).

**Figure 29:**
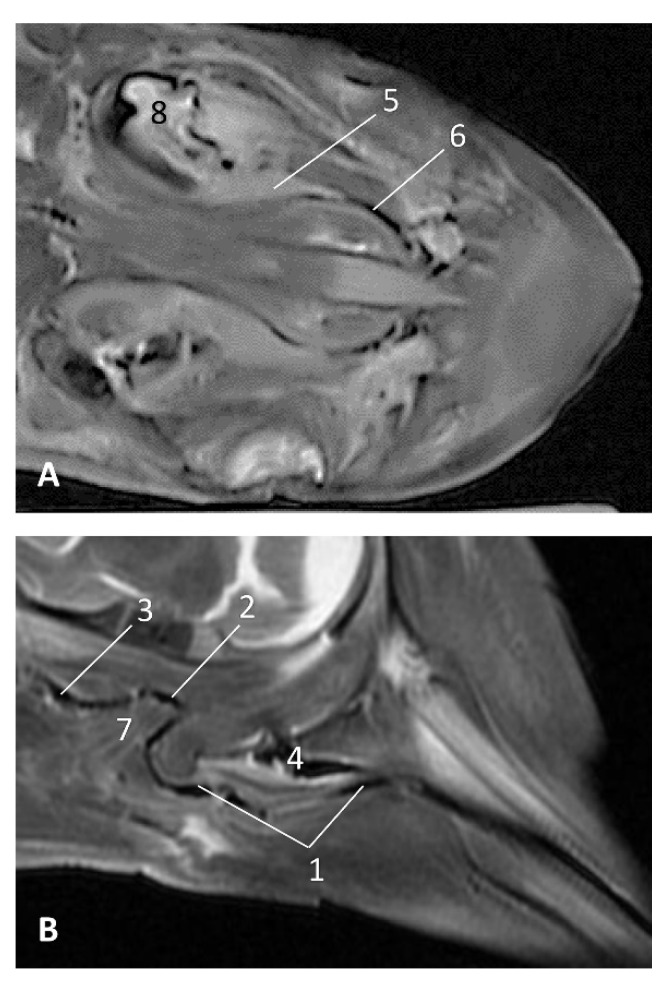
Images of the pharyngeal cavity. (**A**,**B**) MR coronal and sagittal images are oriented so that the rostral is to the right. (**A**,**B**) T2 FrFSE coronal and sagittal planes. 8 months, dde11. 1, Oropharynx: fauces; 2, Nasopharynx; 3, Laringopharynx: oesophageal vestibule; 4, Nasopharynx: pharyngeal diverticulum of the auditory tube; 5, Pharyngeal diverticulum of the auditory tube: hyperintense area (vascular); 6, Pharyngeal diverticulum of the auditory tube: hypointense area (air); 7, Larynx; 8, Middle and inner ear.

**Figure 30:**
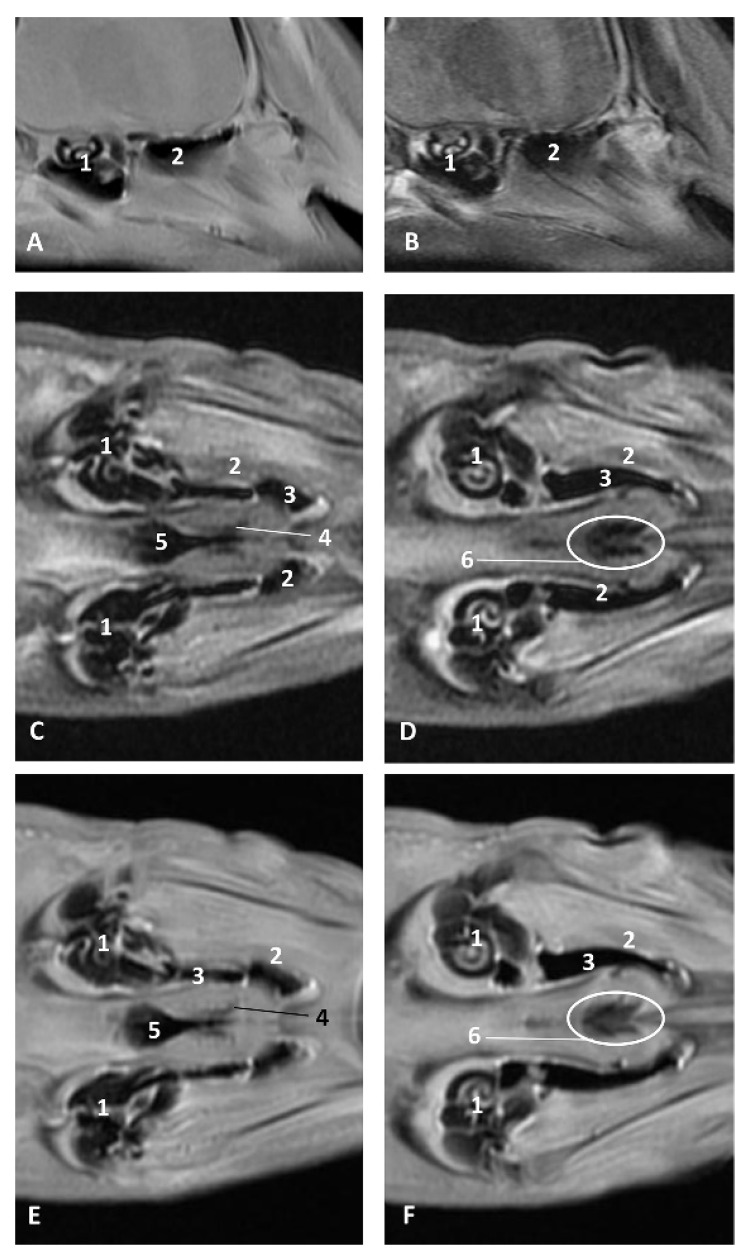
Images of the pharyngeal cavity. MR sagittal and coronal images are oriented so that the rostral is to the right. (**A**) T1 SE sagittal, (**B**) T2 FrFSE sagittal, (**C**,**E**) T1 SE coronal and (**D**,**F**) T2 FrFSE coronal planes. 4 months, dde14. 1, Inner and middle ear; 2, Pharyngeal diverticulum of the auditory tube (vascular); 3, Pharyngeal diverticulum of the auditory tube (air); 4, Auditory tube; 5, Nasopharinx; 6, Intrapharyngeal orifice.

**Figure 31:**
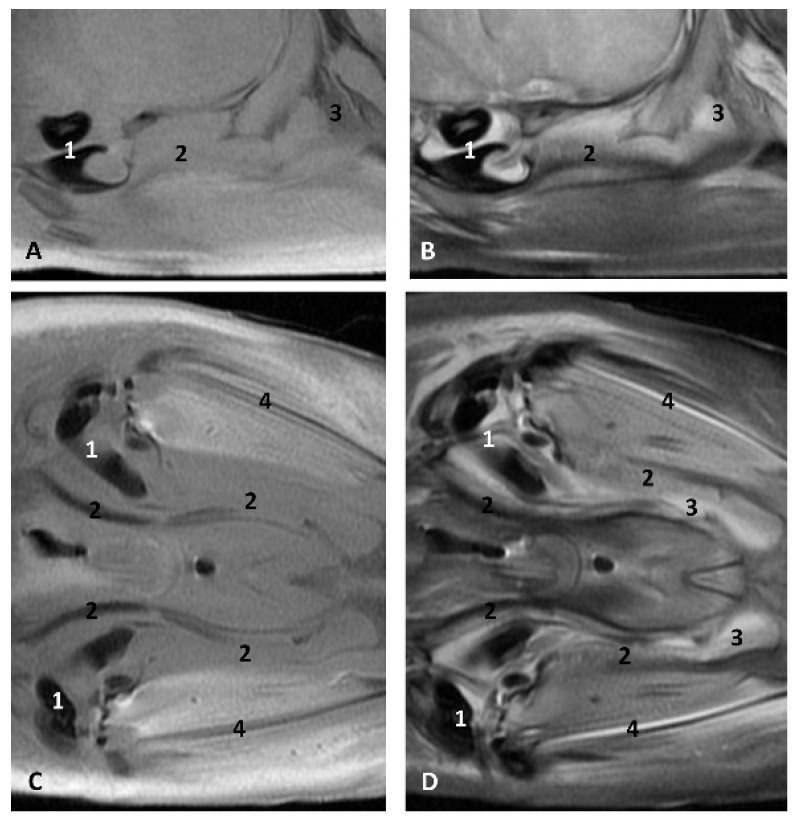
Images of the pharyngeal cavity. MR sagittal and coronal images are oriented so that the rostral is to the right. (**A**) T1 SE sagittal, (**B**) T2 FrFSE sagittal, (**C**) T1 SE coronal and (**D**) T2 FrFSE coronal planes. 9 months, grgr1. 1, Inner and middle ear; 2, Pharyngeal diverticulum of the auditory tube: vascular; 3, Pharyngeal diverticulum of the auditory tube: air; 4, Mandibles.

In older *Delphinus delphis* fetuses (dde5, dde8, dde11) this double space at both sides of the laryngopharynx is more evident and shows the same intensity, but now we can distinguish the vascular area (hyperintense) and the air-filled area (hypointense) (Figure 27, Figure 28, Figure 29, Figure 30 and Figure 31).

In more advanced fetal development, it is possible to observe air (hypointense) and vascular (moderate hyperintense) areas, and even the auditory tube (slightly hypointense) (Figure 30).

PDAT were clearly seen in sagittal and coronal sections in a *Grampus griseus* fetus (grgr1). The T2 sequences are clearer than T1 because they differentiate two areas: slightly hypointense (vascular) and hyperintense (air) (Figure 31).

(b) Histological study

**Figure 32:**
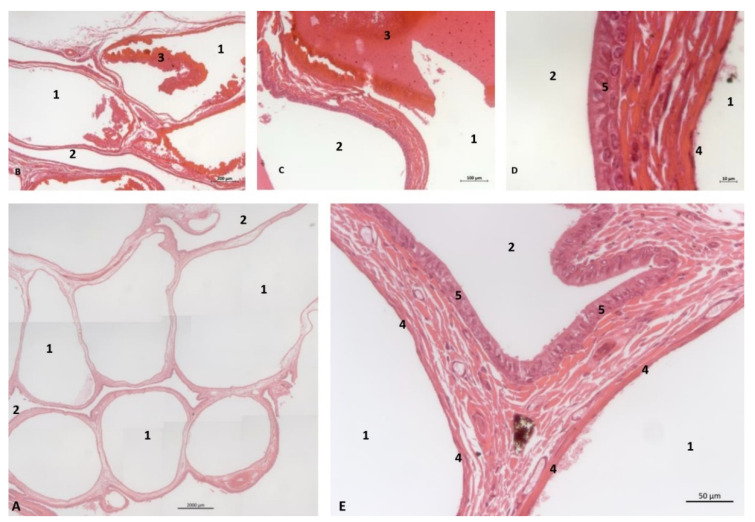
Histological study of the pharyngeal cavity: pharyngeal diverticulum of the auditory tube. (**A**) Pharyngeal vascular plexus wide. (**B**,**C**) Detail of plexus with blood in lumen. (**D**,**E**) Detail of plexus wall. Adult, scomu6. 1, Vascular lumen; 2, Respiratory lumen; 3, Blood vessel lumen; 4, Vascular endothelium; 5, Respiratory epithelium.

The histological analysis of the PDAT shows two well-defined areas inside: a pharyngeal vascular plexus (Figure 32A) with abundant and dilated vascular endothelium and the respiratory epithelium area in contact with air (Figure 32 E). A detailed image reveals the luminal vessels filled with blood (Figure 32B,C). The wall of PDTA is filled with air, which is in contact with respiratory epithelium (Figure 32D,E).

(c) Anatomical study

(c1) Osteology

**Figure 33:**
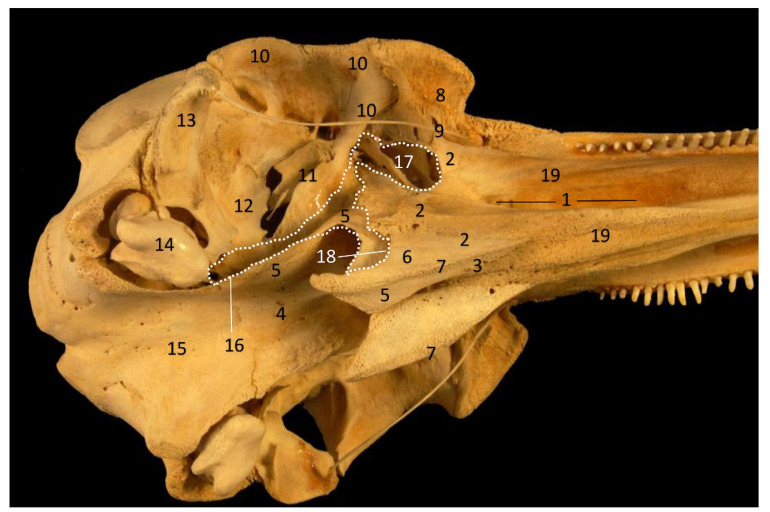
Common dolphin skull. Dots show the extension and form of the right pharyngeal diverticulum of the auditory tube. Photography Francisco Gil Cano. Courtesy from Ángel Tórtola. Spanish naturalist. Oblique view. dde15. 1, Greater palatine groove; 2, Palatine bone: perpendicular lamina; 3, Palatine bone: horizontal lamina; 4, Vomer bone; 5, Pterygoid bone: medial lamina; 6, Pterygoid bone: lateral lamina; 7, Pterygoid bone: crest; 8, Lacrimal and zygomatic bone; 9, Temporal process of the zygomatic bone; 10, Frontal bone; 11, Presphenoid bone: wings; 12; Basisphenoid bone: wings; 13, Temporal bone: squamous part; 14, Temporal bone: petrous and tympanic parts; 15, Occipital bone: basilar part; 16, PDAT area; 17, Maxilopalatine fossa (pterygopalatine fossa in mammals); 18, Pterygopalatyne recess (pterygoid sinus); 19, Maxillary bone: palatine process.

The PDAT is a well delimited area, even in the early stages of fetal development (Figure 2). This area is medially extended to the bony choanae and extends dorsally to the maxillopalatine fossa, medially to the pterygopalatine recess (pterygoid sinus) and rostrally to the petrous and tympanic parts of the temporal bone (cochlea) (Figure 33).

(c2) Sectional anatomy

**Figure 34:**
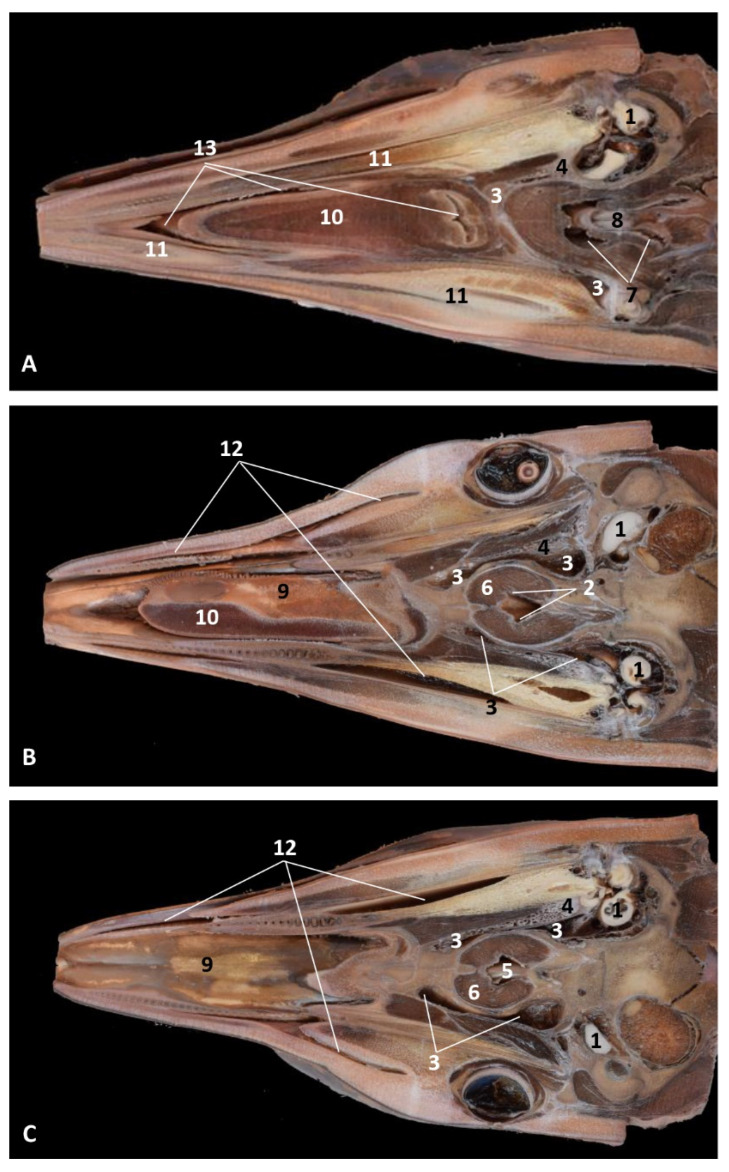
(**A**–**C**) Coronal sections of head at level of eyes, ear, pharyngeal and oral cavity. These three sections show the extension and connection between the pterygopalatine recess (pterygoid sinus) and the PDAT and between the nasopharynx and PDAT. (**A**,**B**) Dorsal view (**C**) Ventral view. scomu2. 1, Middle and inner ear; 2, Pharyngeal orifices of the auditory tube; 3, Pharyngeal diverticulum of the auditory tube: air area; 4, Pharyngeal diverticulum of the auditory tube: vascular area; 5, Vomer and choanas; 6, Pharyngeal muscles; 7, Piriform recess; 8, Laryngeal cartilages: aditus laryngis; 9, Hard palate (maxillary bones); 10, Tongue (sectioned) 11, Mandibles; 12, Labial vestibule; 13, Oral cavity.

The three coronal sections, in a newborn *Stenella coeruleoalba* (scomu2), extend from the floor (Figure 34A) to the roof of the oral and pharyngeal cavities (Figure 34B,C). In these images it was possible to observe the proximity to the mandible channel tissue and the pharyngeal orifices of the auditory tubes crossing the pharyngeal muscles. It is easy to medially differentiate the air area (near the auditory tube and nasopharynx and laterally to the cribriform area) (Figure 34).

**Figure 35:**
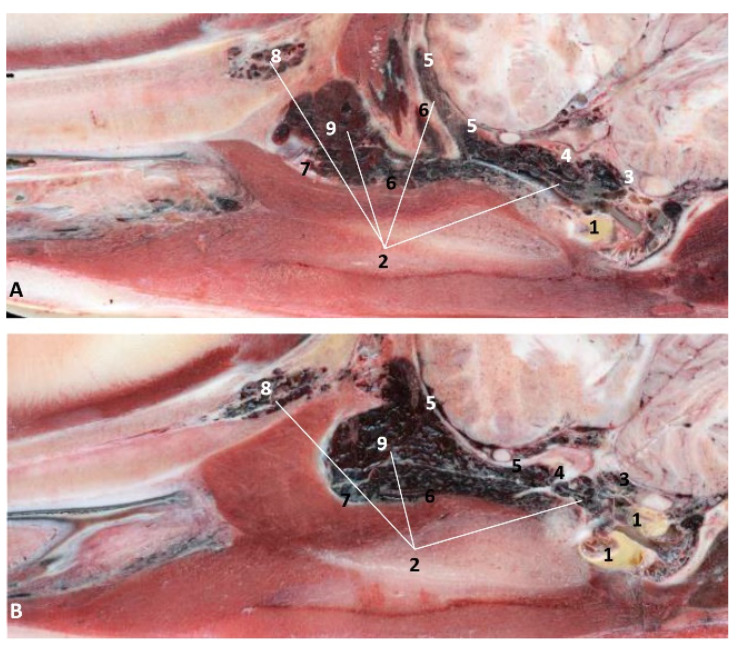
(**A**,**B**) Detailed serial sagittal sections at level of the pharyngeal diverticulum of the auditory tube with an anfractuous mucosa filled with a heterogeneous content. It extends up to the maxillopalatine fossa rostral to the eyeball. scomu3. 1, Middle and inner ear; 2, Pharyngeal diverticulum of the auditory tube; 3, Occipital bone: basilar part; 4, Basisphenoid bone; 5, Presphenoid and ethmoid bones; 6, Pterygoid bone; 7, Palatine bone; 8, Maxilopalatine fossa (pterygopalatine fossa in domestic mammals); 9, Pterygopalatine recess (pterygoid sinus).

The two sagittal sections in a juvenile *Stenella coeruleoalba* (scomu3) were made para-sagittally at the level of the ear. It shows that this area (PDAT) extends rostrally to the inner and middle ear crossing below the basal bones of the cranium to arrive to the pterygopalatine recess (pterygoid sinus) and finish dorsally at the maxillopalatine fossa (Figure 35).

**Figure 36:**
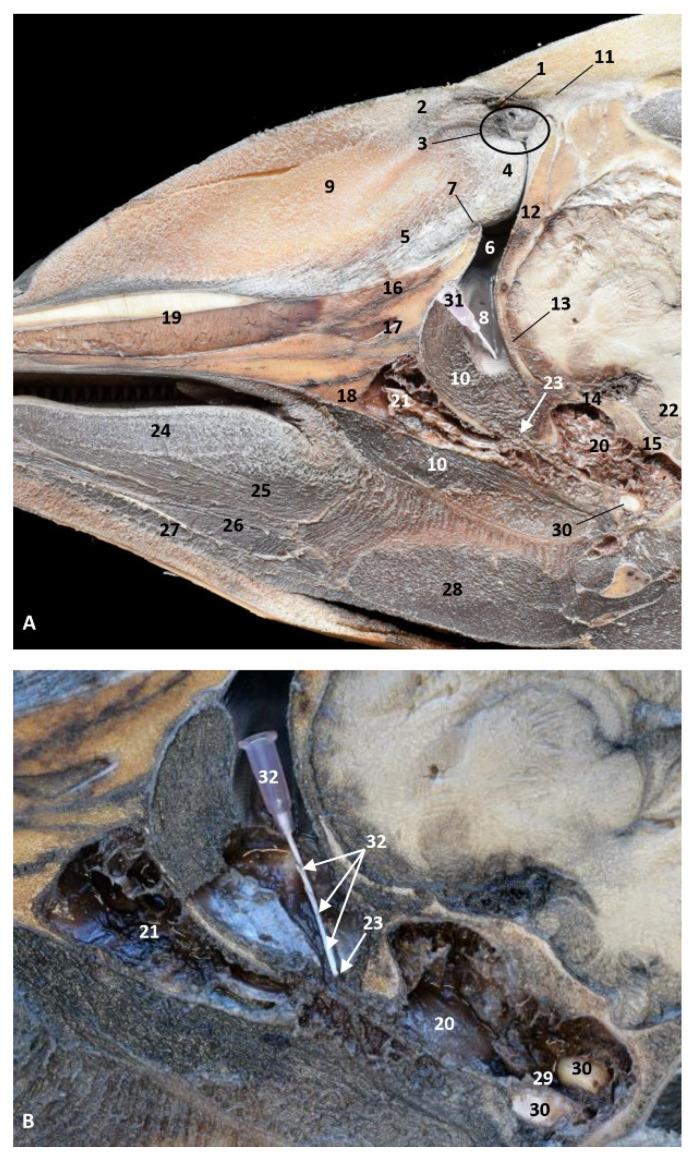
(**A**) Sagittal section of head at level of nasal, pharyngeal and oral cavity. (**B**) Detail of the trajectory of the trocar towards the pharyngeal diverticulum after removing the pharyngeal muscles around the auditory tube. Adult, scomu6. 1, Nasal cavity: vestibule; 2, External nares muscles; 3, Phonic lips; 4, Nasal plug; 5, Nasal plug muscles; 6, Nasal cavity: respiratory part; 7, Nasal cavity: incisive recess; 8, Choanae; 9, Melon; 10, Pharyngeal muscles; 11, Nasal bone; 12, Frontal bone; 13, Ethmoid bone; 14, Presphenoid bone; 15, Basisphenoid bone; 16, Incisive bone; 17, Maxillary bone; 18, Pterygoid bone; 19, Mesethmoid cartilage; 20, Pharyngeal diverticulum of the auditory tube (rostral part is pterygoid sinus); 21, Pterygopalatine recess (pterigoyd sinus); 22, Hypophysis; 23, Connection orifice; 24, Tongue: proper lingual muscle; 25, Hyoglossus muscle; 26, Geniohyoid muscle; 27, Mylohyoid muscle; 28, Digastricus muscle; 29, Musculotubaric channel; 30, Middle ear (petrotympanic bone); 31, Trocar inserted in the pharyngeal orifice of the auditory tube; 32, Trocar (showing duct trajectory).

In the adult *Stenella coeruleoalba* (scomu6), the pharyngeal orifice of the auditory tube is canalized by a trocar (Figure 36A) and the PDAT area is located ventrally. The enlarged image shows, after removing the pharyngeal muscles, the trajectory of the auditory tube towards the pharyngeal diverticulum (Figure 36B).

(c3) Dissection

**Figure 37:**
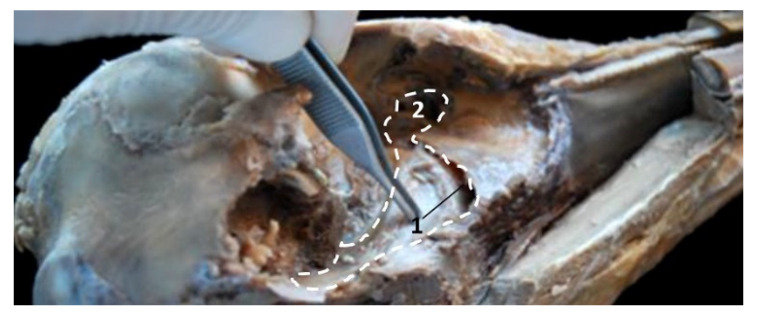
Deep dissection of the dolphin head after removing petrous and tympanic part of the temporal bone. Discontinuous line shows the extension and form of the right pharyngeal diverticulum of the auditory tube. Newborn, scoce1. 1, Pterygopharyngeal recess (pterygoid sinus); 2, Maxilopalatine fossa (pterygopalatine fossa in domestic mammals).

A deep dissection in a newborn *Stenella coeruleoalba* (scoce1) shows the extent of the PDAT area (Figure 37).

## 4. Discussion

### 4.1. Oral Cavity

#### 4.1.1. Vestibule

The oral vestibule in terrestrial mammals could be sub-divided in oral and labial vestibules, due to the presence of a space between lips and teeth (labial vestibule), and between cheeks and teeth (buccal vestibule). In cetaceans, which lack cheeks and, therefore, masseter muscles during development [3], only the labial one is present.

Additionally, in terrestrial mammals, there are superior and inferior labial frenula to capture air and grab food. In cetaceans, the lips are tightly sealed, so the mouth is hermetically closed as it happens with the blowhole. Therefore its lips lack the mobility of the terrestrial mammal. Therefore, the labial vestibule is unique in cetaceans because it lacks a buccal vestibule and it is different from terrestrial mammals.

#### 4.1.2. Oral Cavity Proper

(a) Roof

The rostral part of hard palate has only a vestigial incisive papilla. Since the ontogeny reflects the phylogeny, the ducts are more formed in early stages of development and degenerate as gestation progresses. The innervation observed in this incisive papilla is probably related to the tactile sensitivity to test the mucosa of some prey. This sensation is collected by the major palatine nerve from the maxillary branch of trigeminal nerve.

(b) Tongue

The lingual papillae in the *Globicephala melas*, a teuthophagous (consuming cephalopods) suction feeder [2], may enable its specific feeding strategy, especially when combined with the modifications to its hyoid apparatus, and often to the skull and jaws [28]. On the other hand, *Stenella coeruleoalba* and *Delphinus delphis* dolphin’s lingual papillae do not extend caudally, and these disappear as the animal matures (as in terrestrial mammals).

The regressing papillae may be due to the adult type of feeding and with the prolonged lactation period, 19–20 months in *Stenella coeruleoalba* and *Delphinus delphis* and 24 months or even longer in *Globicephala melas* [26]. Newborn carnivores and suids also have marginal papillae [24].

In birds, there are blade-like lamellae in the inner and outer margin of the bill. For instance, anatids generally have filtering papilla, while ducks have a double row of overlapping bristles in the tongue that interdigitate with a double row of lamellae on the bill [29].

#### 4.1.3. Histological Considerations

Refs. [10,26] show the lingual seromucous and mucous glands in *Delphinus delphis*. We also confirm the presence of mucous glands in an adult *Stenella coeruleoalba* (scomu6) at these levels as well as in the oesophagus, confirmed by the endoscopic images. We also observed mucous glands in *Delphinus delphis* fetus (dde10). The large number of mucous glands has a double function, first to serve as mechanical protection against the abrasive diet, i.e., the fins and scales of fishes and second, to favour lubrication to enable a proper food movement during swallowing. The same conclusions apply to the oropharynx. In addition, the absence of salivary glands explains the abundant mucous glands as a substitute.

In birds, there is a similar compensation process [29], which is of great utility in the digestive process of certain avian species such as galliforms, which ingest seeds, grains and even stones and mud to help trituration.

As for the immune system, the large number of lymphatic vessels (Figure 13) explains the absence of nodular formations (tonsils and nodules) which, in cetaceans, could jeopardize deglutition. The absence of lymphoreticular tissue does not indicate lack of immune function.

The frenulum in most terrestrial animals is wider and more mobile. In dogs and horses, it is sharper and more angled like *Delphinus delphis*, *Stenella coeruleoalba* and *Globicephala melas*. In cetaceans the tongue has limited mobility, serving first during lactation and then during the rest of life, as it is useful to grab food and expel extra water.

The small grooves may, in fact, be the location of the hitherto unveiled taste receptors [10].

We did not locate receptors for taste or olfaction in the oral or nasal cavity [3].

(c) Floor of oral cavity

The sublingual recess and labial vestibule are quite shallow, perhaps due to the absence of mastication, which nullifies the need for a functional space outside the vestibule (outer) surface of the teeth [26].

We see this narrowness of the labial vestibules and sublingual recess during fetal development. Within the lateral sublingual folds, we did not observe the presence of any duct transporting saliva from the major salivary glands, as would happen in terrestrial mammals. Neither did we observe sublingual papillae, as in domestic mammal species.

The monostomatic salivary glands are absent in cetaceans [3]. Stomas opened along the lateral sublingual folds (polystomatic gland) and major sublingual and mandibular ducts, with the latter atrophied in cetaceans, as we have observed in histology (Figure 14).

Even though the mouth is a tight opening, when the dolphin feeds under the water, the entrance of some salt water is unavoidable, but this will be ejected later with the help of the tongue. In a humid environment, the saliva would be unnecessary.

(d) Teeth

Tooth formation occurs below the gums [3]. The final eruption takes place during the perinatal period after lactation (this, along with the few development of the incisive teeth, protects the mother’s nipple), probably by the scratching of the gums against the rough surfaces of food.

In *Delphinus delphis,* teeth are developed just in the molar region; the mandibular teeth develop later. *Grampus griseus* (Figure 31) and *Globicephala melas* (Figure 7) have fewer teeth due to their shorter and rounder jaw. In this last species, the caudal teeth do not develop, since the mandible grows and the teeth remain rostral in position. In *Phocoena phocoena* (phog1) the conic teeth (Figure 2) with which they are born will flatten and round with age by the process of wear.

For the first time, we discovered that the teeth, during development, are covered by cementum under the gums. We can state that odontocete teeth are phylogenetically closer to the hypsodont dentition of ruminants and equids, in which teeth are composed from the crown to root as follows: a core of dentine covered internally and externally by enamel which is, in turn, covered by cementum (Figure 12d and Figure 13d).

Cetacean teeth are designed to grab and swallow but not to chew, so molars are not necessary.

### 4.2. Pharyngeal Cavity

During fetal development, we observed that the soft palate of odontocetes stays attached caudally along the crest of the pterygoid bone (Figure 13), while in terrestrial mammals, it remains attached to the horizontal lamina of the palatine bone (palatine aponeurosis).

The isthmus of the fauces is tightly closed, as happens with the blowhole [3], because it is formed by musculomembranous walls, like those of the oesophagus.

The tightly closed oropharynx (Figure 12) can be opened (Figure 15) during gestation to allow the entrance of amniotic fluid into the fetal alimentary canal to stimulate the glands producing gastric enzymes [30,31]. Once born, neither the oropharynx nor the oesophagus open until food is swallowed.

The endoscope overcomes the resistance of the isthmus of the fauces by exerting pressure similar to the effect produced by the ingestion of prey.

(a) Histological considerations

The lateral asymmetry in dolphins exists to the right externally, and to the left internally. [28] This defends a trophic explanation since the internal asymmetry allows the ingestion of larger prey, though we believe the asymmetry described is the opposite, since it is not the right piriform recess but the left one which is the larger (Figure 21). The voluntary dislocation of the larynx to ingest larger prey has already been described [32].

Nasopharynx

The pharyngeal orifice of the auditory tube is an open membranous duct (Figure 36a) close to the pharyngeal muscles. The PDAT connects to the musculotubaric channel and finishes in the tympanic orifice of the auditory tube.

The pterygopalatine recess (pterygoid sinus) is not isolated but connected to the pharyngeal diverticulum of the auditory tube at the level of the auditory tube notch [33]). We do not consider the pterygopalatine recess as an isolated space of the auditory tube diverticulum, but instead as part of the structure.

In the horse, the auditory tube diverticulum extends from the skull base to the atlas dorsally and ventrally to the pharyngeal cavity roof [34,35].

The fact that the auditory tube is not a closed duct but dilating and folding according to pressure, has its application in decompression. The auditory tube protects from threats (i.e., strong sounds which are potentially dangerous to the small bones) forcing the dolphin to emerge quickly releasing high-pressure air (or liquid) through the orifice. This theory can be reinforced by the fact that the Vater–Paccini corpuscles (Figure 24) are pressure receptors observed in the nasopharyngeal mucosa close to the pharyngeal orifice of the auditory tube (Figure 36).

This would also prevent the collapse of the tracheal and respiratory walls while simultaneously ensuring that the air at great depths is distributed to essential areas.

The blood present can have a double function of refrigeration and a reservoir of extra oxygen, available for exchange with blood vessels in part of the epithelium and within the sinuses. It could be very interesting to further examine this process in Mysticetes. A similar process happens in other parts of the lower respiratory system [36].

(b) Comparative Anatomy

In the horse, the function of these diverticula is to cool the blood going to the brain via the internal carotid and the external carotid artery. In equids, the diverticula are divided by a membranous septum extending to the ventral face of the atlas. In odontocetes, it extends only to the pterygopalatine recess (pterygoid sinus) and medially to the pterygoid bone. The opposite happens in terrestrial mammals (where the diverticulum of the auditory tube is divided by the basilar portion ridge, the basihyoid, and the medial lamina of the pterygoid bone [34] and [35]. Both diverticula are divided by bone, so the entrance to the larynx (aditus laryngis) is not interrupted at this level.

(c) Histological considerations

The deep serosal glands observed in the nasopharynx secrete towards the small openings observed in the deepest area of the nasal cavity [3] and to the small holes observed in the nasopharynx area close to the pharyngeal orifice of the auditory tube (Figure 21, Figure 22 and Figure 23). Close to these orifices, a pressure corpuscle (Figure 24) was observed. This possibly indicates that, when the vascular plexus observed in the pharyngeal diverticulum of the auditory tube is filled with blood (Figure 27, Figure 28, Figure 29, Figure 30, Figure 31 and Figure 32 and 34–36), this process promotes the expulsion of the air stored to the auditory tube into the nasopharynx (Figure 36). The air pressure within the nasopharynx is detected by the Vater–Paccini corpuscles (Figure 24).

## 5. Conclusions

Inside the oral cavity proper, incisive papilla are present in all specimens to test food texture and hardness and, during lactation, this is also reinforced by the lack of incisive teeth to prevent nipple harming. Comparatively, we can state that *Delphinus delphis* palate is morphologically different from the other species due to an additional groove.

We have also seen the form and function of the tongue showing the marginal papillae disappearing gradually after lactation, lasting more in some species like *Globicephala melas*. The teeth in formation present three layers under the gum, developed during fetal life but erupting through the gums after lactation.

We found mucous glands in the oral and pharyngeal cavities, with a double function of lubrication and mechanical protection. In the nasopharynx, we found serous glands to humidify the area as well as pressure corpuscles.

In the laryngopharynx, we realized that the left piriform recess was larger than the right one, probably to allow the ingestion of larger prey.

In the nasopharynx, one of the achievements of this study was finding and visualizing, for the first time, the diverticula of the auditory tube from early stages of the development, using MRI and the dissection of fetus skulls. This space extends rostrodorsally from the ear to the nasopharynx and contains air and a vascular plexus, similar to those mammals which possess such a structure.

Buccopharyngeal cavity endoscopies would also be useful to highlight alterations in the anatomical structures at these levels, creating situations incompatible with life.

## Figures and Tables

**Table 1:** Specimens of dolphin used in this study. dde: *Delphinus delphis* (Linnaeus 1758) from Galicia, Spain; scop: *Stenella coeruleoalba* (Meyen 1833) from Galicia, Spain; gma: *Globicephala melas* (Traill 1809) from Galicia, Spain; scoce: *Stenella coeruleoalba* (Meyen 1833) from Ceuta, Spain; scomu: *Stenella coeruleoalba* (Meyen 1833) from Murcia, Spain; phog: *Phocoena phocoena* (Linnaeus 1758) from Galicia, Spain; grgr: *Grampus griseus* (Cuvier 1912) from Valencia, Spain; MRI: Magnetic resonance imaging.

Study Code	Specie, Sex Stage [25,26,27].	Anatomical, Surgical/Imaging Diagnostic Techniques
dde1	*Delphinus delphis* L, male fetus	Endoscopy
dde2	*Delphinus delphis* L, male fetus	Endoscopy
dde3	*Delphinus delphis* L, female fetus	Endoscopy, MRI
scop1	*Stenella coeruleoalba* M, female fetus	Endoscopy
gma1	*Globicephala melas* T, male fetus	Endoscopy, MRI
dde5	*Delphinus delphis* L, female fetus	Endoscopy, MRI
dde6	*Delphinus delphis* L, female fetus	Endoscopy
dde7	*Delphinus delphis* L, male fetus	Photography
dde8	*Delphinus delphis* L, female fetus	Endoscopy. MRI
dde9	*Delphinus delphis* L, male fetus	Endoscopy
dde10	*Delphinus delphis* L, female fetus	Endoscopy, histological analysis
dde11	*Delphinus delphis* L, male fetus	Endoscopy, MRI
dde12	*Delphinus delphis* L, male fetus	Endoscopy
dde13	*Delphinus delphis* L, female fetus	Endoscopy, MRI
phog1	*Phocoena phocoena* L, female fetus	Osteology
dde14	*Delphinus delphis* L, female fetus	Endoscopy, MRI, histological analysis
scoce1	*Stenella coeruleoalba* M, male newborn	Head dissection
scomu1	*Stenella coeruleoalba* M, female newborn	Oral cavity analysis
scomu2	*Stenella coeruleoalba* M, male newborn	Head coronal section
grgr1	*Grampus griseus* C, female fetus	MRI
scomu3	*Stenella coeruleoalba* M, male juvenile	Head sagittal section
scomu4	*Stenella coeruleoalba* M, Male juvenile	Endoscopy
scomu6	*Stenella coeruleoalba* M, male adult	Head sagittal section, histological analysis
dde15	*Delphinus delphis* L, adult	Osteology

## Data Availability

Not applicable.

## Supplementary Materials

The following are available online at https://www.mdpi.com/article/10.3390/ani11061507/s1, Table S1: Other parameters observed in this study. Table S2: MRI parameters used in this study.
