# Supplementary material for: Endoscopic Study of the Oral and Pharyngeal Cavities in the Common Dolphin, Striped Dolphin, Risso’s Dolphin, Harbour Porpoise and Pilot Whale: Reinforced with Other Diagnostic and Anatomic Techniques
Source: Animals (Basel). 2021 May 22;11(6):1507. doi: 10.3390/ani11061507 (PMC8224762; doi:10.3390/ani11061507)
Supplement: Supplementary file 1 [file animals-11-01507-s001.zip › Table S1.pdf]

Table S1. Other parameters observed in this study.

Stranding Reference	Length, Weight, Estimated Gestation Time and Stranding Year [25,26,27]	Preservation Techniques
DDE1, CEMMA	11.8 cm, 15.4 gr, 1,5 months approx., 2012.	Fixation: formaldehyde 10% and refrigeration
DDE2 CEMMA	27.5 cm, 212.8 gr, 3,5 months approx., 2012.	Fixation: formaldehyde 10% and refrigeration
DDE3 CEMMA	31 cm, 340.4 gr, 4 months aprox., 2014.	Fixation: formaldehyde 10% and refrigeration
SCOP1 CEMMA	32.5 cm, 372 gr, 4.5 months approx., 2004.	Fixation: formaldehyde 10% and refrigeration
GMA1 CECAM	40 cm, 628.3 gr, 5 months approx., 2013.	Fixation: formaldehyde 10% and refrigeration
DDE4 CEMMA	41.5 cm, 842.3 gr, 5.5 months approx., 2014.	Fixation: formaldehyde 10% and refrigeration
DDE5 CEMMA	41.7 cm, 655.3 gr, 5.5 months approx., 2009.	Fixation: formaldehyde 10% and refrigeration
DDE6 CEMMA	44 cm, 864.5 gr, 5.8 months approx., 2008.	Fixation: formaldehyde 10% and refrigeration
DDE7 CEMMA	50 cm, 1061.4 gr, 6 months approx., 2009.	Fixation: formaldehyde 10% and refrigeration
DDE8 CEMMA	47.5 cm bis, 948.9 gr, 6 months approx., 2014.	Fixation: formaldehyde 10% and refrigeration
DDE9 CEMMA	50.5 cm, 1037.4 gr, 7 months approx., 2012.	Fixation: formaldehyde 10% and refrigeration
DDE10 CEMMA	50.5 cm, 1231.1 gr, 7.5 months approx., 2014.	Fixation: formaldehyde 10% and refrigeration
DDE11 CEMMA	56 cm, 1470 gr, 8 months approx., 2009.	Fixation: formaldehyde 10% and refrigeration
DDE12 CEMMA	58 cm, 1483.9 gr, 8,5months approx., 2008.	Fixation: formaldehyde 10% and refrigeration
DDE13 CEMMA	65 cm, 3090 gr, 9 Months approx., 2004.	Embalming: formaldehyde, glycerine, isopropyl alcohol, phenol and refrigeration
PHOG1 OVA	74 cm, 9 months approx., 2010	Necropsy
DDE14 CEMMA	75 cm, 3110 gr, 10 months, 2014.	Embalming: formaldehyde, glycerine, isopropyl alcohol, phenol and refrigeration
SCOCE1 CECAM	85 cm, 9200 gr, 2014.	Fixation: formaldehyde 10%
SCOMU1 CRFS	96 cm, 10.840 gr, 2017.	Fixation: formaldehyde 10% and freezing - 20ºC
SCOMU2 CFRS	102 cm, 14.000 gr, 2012.	Fixation: formaldehyde 10% and refrigeration

GRGR1<br>OVA	106 cm, 8570 gr., 13 months, 2016	Necropsy
SCOMU3<br>CFRS	123 cm, 23.650 gr., 2019.	Fixation: formaldehyde 10% and refrigeration
SCOMU4<br>CFRS	176 cm, 54,900 gr, 2019.	Freezing - 20ºC
SCOMU6<br>CFRS	201 cm, 83.850 gr, 2018.	Embalming: formaldehyde, glycerine, isopropyl alcohol, phenol and refrigeration
DDE15<br>PC	Cranium	Room temperature

DDE: Delphinus delphis from Galicia, Spain; SCOP: Stenella coeruleoalba from Galicia, Spain; SCOCE: Stenella coeruleoalba from Ceuta, Spain; SCOMU: Stenella coeruleoalba from Murcia, Spain; MRI: Magnetic resonance imaging; CT: Computed Tomography, CEMMA: Coordinator Center for the study of the marine mammals, Galicia, Spain; CECAM: Center for the study and conservation of marine animals, Ceuta, Spain; CRFS: Wildlife rehabilitation Center, Murcia, Spain; OVA: Oceanographic, Valencia, Spain; PC: Private Collection, Murcia, Spain.
