# Supplementary material for: Endoscopic Study of the Oral and Pharyngeal Cavities in the Common Dolphin, Striped Dolphin, Risso’s Dolphin, Harbour Porpoise and Pilot Whale: Reinforced with Other Diagnostic and Anatomic Techniques
Source: Animals (Basel). 2021 May 22;11(6):1507. doi: 10.3390/ani11061507 (PMC8224762; doi:10.3390/ani11061507)
Supplement: Supplementary file 1 [file animals-11-01507-s001.zip › Table S2.pdf]

Table S2. MRI parameters used in this study.

Study Code	Weighted	Pulse Sequence	Dimensional Plane	Acquisition	TE	TR	TI	NEX	Slice Thickness	Interslice Gap	Field of View	Matrix Dimensions
dde3	T1	se	sagittal	2D	14	360	0	3	3	3.3	100	0\320\224\0
dde3	T2	frfse	sagittal	2D	104	2000	0	3	3	3.3	100	0\320\224\0
dde3	T1	se	coronal	2D	10	420	0	3	3	3.3	100	0\320\224\0
dde3	T2	frfse	coronal	2D	104	2500	0	3	3	3.3	100	0\320\224\0
gma1	T1	se	sagittal	2D	14	400	0	3	3	3.3	100	0\320\224\0
gma1	T2	frfse	sagittal	2D	104	2000	0	2	3	3.3	100	0\320\224\0
gma1	T1	se	coronal	2D	10	540	0	3	3	3.3	100	0\320\224\0
gma1	T2	frfse	coronal	2D	104	3240	0	3	3	3.3	100	0\320\224\0
dde5	T2	frfse	sagittal	2D	104	2000	0	3	3	3.3	100	0\320\224\0
dde5	T1	se	coronal	2D	10	260	0	3	3	3.3	100	0\320\224\0
dde5	T2	frfse	coronal	2D	104	3000	0	3	3	3.3	100	0\320\224\0
dde8	T1	se	sagittal	2D	14	300	0	3	3	3.3	100	0\320\224\0
dde8	T2	Frfse	Sagittal	2D	104	3000	0	3	3	3.3	100	0\320\224\0
dde8	T1	se	coronal	2D	10	300	0	3	3	3.3	100	0\320\224\0
dde8	T2	Frfse	coronal	2D	104	1760	0	3	3	3.3	100	0\320\224\0

dde11	T1	frfse	sagittal	2D	104	2000	0	3	3	3.3	100	0\320\224\0
dde11	T2	frfse	coronal	2D	104	3500	0	3	3	3.3	100	0\320\224\0
dde13	T1	se	sagittal	2D	14	300	0	3	4	4.1	75	0\320\224\0
dde13	T2	frfse	sagittal	2D	105	3820	0	2	4	4.1	100	384\0\0\224
dde13	T1	se	coronal	2D	14	240	0	2	3.5	3.8	75	0\320\256\0
dde13	T2	frfse	coronal	2D	92	3200	0	2	3.5	3.8	75	0\320\224\0
dde14	T1	se	sagittal	2D	14	300	0	3	4	4.1	75	0\320\224\0
dde14	T2	frfse	sagittal	2D	107	3500	0	2	4	4.1	100	384\0\0\224
dde14	T1	se	coronal	2D	14	460	0	2	3.5	3.8	75	0\320\256\0
dde14	T2	frfse	coronal	2D	94	4040	0	2	3.5	3.8	75	0\320\224\0
grgr1	T1	se	sagittal	2D	14	560	0	2	3	3.3	100	0\320\224\0
grgr1	T2	frfse	sagittal	2D	104	2000	0	2	3	3.3	100	0\320\224\0
grgr1	T1	se	coronal	2D	10	500	0	2	3	3.3	100	0\320\224\0
grgr1	T2	frfse	coronal	2D	104	2240	0	2	3	3.3	100	0\320\224\0

se: Spin echo sequence; *FrFse:* Fast Recovery Fast Spin Echo sequence.
